# Degradation and Stabilization of Peptide Hormones in Human Blood Specimens

**DOI:** 10.1371/journal.pone.0134427

**Published:** 2015-07-29

**Authors:** Jizu Yi, David Warunek, David Craft

**Affiliations:** BD Diagnostics, One Becton Drive, Franklin Lakes, NJ, United States of America; University of Würzburg, GERMANY

## Abstract

Plasma hormone peptides, including GLP-1, GIP, Glucagon, and OXM, possess multiple physiological roles and potential therapeutic and diagnostic utility as biomarkers in the research of metabolic disorders. These peptides are subject to proteolytic degradation causing preanalytical variations. Stabilization for accurate quantitation of these active peptides in *ex vivo* blood specimens is essential for drug and biomarker development. We investigated the protease-driven instability of these peptides in conventional serum, plasma, anticoagulated whole blood, as well as whole blood and plasma stabilized with protease inhibitors. The peptide was monitored by both time-course Matrix-Assisted Laser Desorption Ionization Time-to-Flight Mass Spectrometry (MALDI –TOF MS) and Ab-based assay (ELISA or RIA). MS enabled the identification of proteolytic fragments. In non-stabilized blood samples, the results clearly indicated that dipeptidyl peptidase-IV (DPP-IV) removed the N-terminal two amino acid residues from GLP-1, GIP and OXM(1-37) and not-yet identified peptidase(s) cleave(s) the full-length OXM(1-37) and its fragments. DPP-IV also continued to remove two additional N-terminal residues of processed OXM(3–37) to yield OXM(5–37). Importantly, both DPP-IV and other peptidase(s) activities were inhibited efficiently by the protease inhibitors included in the BD P800* tube. There was preservation of GLP-1, GIP, OXM and glucagon in the P800 plasma samples with half-lives > 96, 96, 72, and 45 hours at room temperature (RT), respectively. In the BD P700* plasma samples, the stabilization of GLP-1 was also achieved with half-life > 96 hours at RT. The stabilization of these variable peptides increased their utility in drug and/or biomarker development. While stability results of GLP-1 obtained with Ab-based assay were consistent with those obtained by MS analysis, the Ab-based results of GIP, Glucagon, and OXM did not reflect the time-dependent degradations revealed by MS analysis. Therefore, we recommended characterizing the degradation of the peptide using the MS-based method when investigating the stability of a specific peptide.

## Introduction

In human circulation, peptide hormones, such as glucagon-like peptide 1 (GLP-1), glucose-dependent insulinotropic polypeptide (GIP), oxyntomodulin (OXM) and glucagon, play multiple physiological roles [1, 2]. As such, these peptides or their analogs have attracted extensive research activities to develop therapeutic applications for diabetes and obesity. GLP-1 and GIP are two major human “incretin” hormones, which bind to the GLP-1 receptor and stimulate insulin release in a glucose-dependent manner known as the “incretin effect” [3–5]. GLP-1 and GIP contribute to approximately 60–70% of the total postprandial insulin response in healthy individuals, and have a potentially therapeutic value in the treatment of type II diabetes [6–8]. GLP-1 also regulates cell proliferation, differentiation and apoptosis, and has a physiological role in controlling energy homeostasis and balance through both peripheral signals and brain stem regulations of appetite in the nucleus of the solitary tract (NTS) [2, 9]. OXM as a GLP-1 receptor agonist can enhance the “incretin effect” [10], and reduces body weight in human studies by suppressing appetite and reducing food intake [9, 11]. Interestingly, a synthetic GIP-OXM hybrid peptide acting through GIP, glucagon, and GIP receptors exhibits both weight-reducing and anti-diabetic properties [12]. On the other hand, the physiological role of glucagon is to maintain euglycemia during the fasting state by inducing hepatic glucose production in the liver [13]. The peptide is used clinically to raise blood glucose levels in the treatment of hypoglycemia [14]. Furthermore, a clinical study also showed that the ratio of glucagon/insulin in blood levels could be used as a potential biomarker to differentiate type 2 diabetes mellitus from pancreatic cancer-related diabetes mellitus, suggesting that the measurement of the glucagon/insulin ratio might improve early diagnosis in a subset of patients with new onset diabetes [15].

Active GLP-1 has two forms; each differs in their C-terminal ends: a 30–amino-acid (A.A.) residue peptide with the C-terminal end amidated (GLP-1 (7-36A), noted as G36A) and a 31–A.A. peptide with the C-terminal end extended with a glycine (Gly) residue (GLP-1(7–37) noted as G37) (Table 1). Glucagon is a 29-A.A. peptide that has the same sequence but eight C-terminal residues less as compared with oxyntomodulin (1–37) noted as OXM (Table 1). Two GLP-1 forms, glucagon, and OXM are derived by proteolytic cleavages of the same larger protein called proglucagon. These peptides are secreted by L cells located predominantly in the distal gastrointestinal (GI) tract (GLP-1) or intestine (OXM) [3–5, 16], and by alpha cells (α-cells) located in the endocrine portion of the pancreas (glucagon). GIP, a 42-A.A. peptide (Table 1), is derived proteolytically from the ProGIP protein, and is secreted by endocrine K cells that are mainly present in the proximal gastrointestinal (GI) tract[3–5, 16]. Once secreted, all of the four gut peptides can be translocated to and circulate in the blood stream.

**Table 1:** Peptides: their sequences and molecular weights observed in MALDI-TOF MS.

Peptide	Sequence^a^	MH^+^ (Da)^b^
**GLP-1(7–37), G37**	HA/EGTFTSDVSSYLEGQAAKEFIAWLVKGRG	**3355.27**
**GLP-1(7-36A), G36A**	HA/EGTFTSDVSSYLEGQAAKEFIAWLVKGR-NH_2_	**3297.24**
**AQUA GLP-1, AG36**	HA/EGTFTSDVSSYLEGQAAKEFIAWLVKGR-OH	**3306.25**
**GIP(1–42), GIP**	YA/EGTFISDYSIAMDKIHQQDFVNWLLAQKGKKNDWKHNITQ	**4981.7**
**Glucagon**	HS/QGTFTSDYSKYLDSRRAQDFVQWLMNT	**3482.53**
**AQUA Glucagon**	HS/QGTFTSDYSKYLDSRRAQDFVQWLMNT	**3492.54**
**OXM(1–37), OXM**	HS/QGTFTSDYSKYLDSRRAQDFVQWLMNTKRNRNNIA	**4450.08**
**Peptide YY, PYY**	YPIKPEAPGEDASPEELNRYYASLRHYLNLVTRQRY	**4306.4**

a-Two active GLP-1, Aqua GLP-1, GIP, Glucagon and Aqua Glucagon, OXM(1–37) and PYY(1–36) are listed. The underline indicates the labeled residue with stable isotopic ^13^C and ^15^N. DPP-IV cleavage site is indicated “/” when applicable. AQUA peptide incorporates one stable isotope labeled amino acid, creating a slight increase (6–10 daltons) in molecular weight.

b-Monoisotopic peak position observed in MALDI-TOF MS.

The success of peptide-based drug and/or biomarker development, including pharmacokinetic and pharmacodynamic studies, therapeutic monitoring, as well as biomarker development for diagnosis and prognosis of many diseases, is largely dependent on the accurate measurement of the peptide via *ex vivo* specimens such as blood and cerebrospinal fluid. Endogenous peptides can be modified during and post collection by various mechanisms such as degradation, deamidation, and oxidation [13, 17, 18] leading to erroneous results. Similarly, peptide hormones are subject to preanalytical variability during *ex vivo* sample collection and handling [19–22] specifically due to the intrinsic proteolysis [22–24]. The proteolytic degradation causes a sequential multi-step reaction (SMSR) in the digestion of plasma proteins and peptides [23] and significantly increases the sample-to-sample variability [24]. Such preanalytical variability may override a disease-related pattern or suppress meaningful data interpretation [25–27]. This inaccuracy may contribute to the challenge of translating biomarkers from discovery to the clinic [20, 22, 28]. Numerous publications have illustrated that after secretion circulating GLP-1, GIP, OXM and glucagon are rapidly digested by dipeptidyl peptidase-IV (DPP-IV, or CD26) [29–31]. Very short half-lives for these peptides in circulation have been noted: approximately 2 minutes for active GLP-1, 5 minutes for intact GIP [3, 29], 6–8 minutes for OXM [32], and 2–5 minutes for glucagon [32, 33]. Therefore, it is critical to stabilize these peptides at the point of collection through the preanalytical phase to ensure an accurate measurement.

Herein, we investigated the instability of the aforementioned hormone peptides in conventional blood collection tubes, and their stabilization through the use of protease-inhibitors (PIs). The targeted peptides, including active G37, G36A, GIP, Glucagon and OXM (Table 1), were incubated with whole blood, plasma, or serum for specific time intervals, followed by analysis with Matrix-Assisted Laser Desorption Time-of-Flight Mass Spectrometry (MALDI-TOF MS) and antibody-based (Ab-based) immunoassay (ELISA or EIA). Through mass spectrometry (MS), we clearly identified that the fast degradation of these peptide hormones was primarily due to DPP-IV cleavage of the N-terminal two residues. MS also determined that full-length (fl) OXM was cleaved by unidentified proteases or peptidases in traditional EDTA plasma samples. We further showed that the stability of the studied peptides were largely dependent on the sample type. The addition of DPP-IV and other protease inhibitors contained in BD P800 and P700 (BD, Franklin Lakes, NJ) significantly increased their stability.

## Methods

Human blood samples in this study were collected under BD PAS Associate Sample Collection Protocol (CLN-004) approved by Allendale Investigational Review Board (IRB), located at 30 Neck Road, Old Lyme, CT 06371. Tel: 1-860-434-5872. All subjects completed the Informed Consent process and signed the IRB approved Consent Form prior to participation.

### Blood Collection and Plasma/Serum Preparation

Human blood from healthy subjects was directly collected into BD P800 (2.0 mL or 8.5 mL, optimized for the stabilization of GLP-1, GIP, glucagon, and oxyntomodulin), P700 (optimized for GLP-1 stabilization only), K_2_EDTA or serum tubes (BD, Franklin Lakes, NJ) by venipuncture. For whole blood, the tests were carried out right after collection. For plasma samples, tubes were centrifuged for 15 minutes at 2,500 x g (RCF) and at room temperature (RT) and processed immediately as described in previous reports [22, 34]. For serum samples, the tubes were allowed to clot at RT for 60 minutes and then centrifuged under the same conditions as for plasma tubes. The collected plasma and serum samples were either used immediately or frozen immediately at -80°C until later usage.

### Peptides

The hormone peptides used in this study (Table 1) included three GLP-1 isoforms [GLP-1 (7–36) amide (**G36A**), AQUA GLP-1 (7–36) (**AG36**), and GLP-1 (7–37) (**G37**)], one full-length (fl) GIP (1–42), two glucagon peptides (fl glucagon and AQUA glucagon), fl OXM(1–37) (OXM) and full length Peptide YY (PYY). Each peptide was purchased from Sigma-Aldrich Corp. (St. Louis, MO) with the exception of OXM with Arg^33^, which was purchased from Phoenix Pharmaceuticals, Inc. (Burlingame, CA). The primary structure of OXM is identical in all mammals, except for Lys^33^ in pigs and cattle replaced by Arg^33^ in humans and rats. This residue change does not modify the biological properties of OXM [35]. AG36 and AQUA glucagon were synthesized by Sigma-Genosys (The Woodland, Texas) with Lys (K^20^) in the former and Arg (R^17^) in the latter peptides labeled with stable isotopic ^13^C_6_ and ^15^N_4_. The stable isotopic labeled peptides displayed an approximately10 Da (m/z) higher than their natural counterparts in MS (Table 1), and were used as internal controls for MS-based quantitation. PYY was used as a control peptide for GIP.

### Sample Preparation for Time-course MALDI-TOF Mass Spectrometric Analysis

The detailed method for peptide stability investigation by time-course MS has been described in previous reports [23, 36]. Briefly, into 10 μL of either plasma, serum, or whole blood sample pooled from the same three healthy subjects, 1 μL of either GLP-1, or GIP, or glucagon, or OXM-K^33^ solution was spiked to a final concentration of ~ 400 fmol/μL (0.4 μM). The spiked samples were incubated in a temperature-controlled chamber at RT (25 ± 1°C) or on ice. At specific time intervals from 0 up to 96 hours, the whole blood specimens were centrifuged to obtain plasma samples. the plasma or serum samples were quenched by adding acetonitrile (ACN) (Sigma-Aldrich), trifluoroacetic acid (TFA) (T.B. Baker, Phillipsburg, NJ), or acetone (Sigma-Aldrich) to a final concentration of 10% ACN and 0.2% TFA for GLP-1 analysis, or 50% ACN, 30% acetone and 0.2% TFA for GIP, glucagon, and OXM analysis. For a semi-quantitative measurement, an appropriate control peptide was also included in the quenching solution. GLP-1 peptides were extracted from the quenched samples using a Zip-Tip C18 (EMD Millipore, Billerica, MA) followed by MS analysis. The quenched GIP, glucagon and OXM samples were centrifuged under 125,000 x g for 20 minutes to remove precipitated proteins by pelleting. The clear supernatants were dried in a CentriVap Concentrator (Labconco, Kansas City, MO); the dried powders were then re-suspended in 10% ACN, 0.1% TFA solution, followed by Zip-Tip C18 extraction. A 1.0 μL aliquot of the eluted peptide solution was mixed with 1.0 μL of α-Cyano-4-hydroxycinnamic acid (CHCA:10 mg/mL in 50% ACN solution) as the matrix, and 1.5 μL mixture was spotted onto a MALDI target, air-dried, and analyzed by MALDI-TOF MS (Ultraflex II, Bruker Daltonics Inc., Billerica, MA) [36].

### Time-Course Ab-Based Assays

Sample preparation procedures for Ab-based stability studies were similar to those in the time-course MALDI-TOF MS analysis. The peptides were spiked within the linear range of the assays, with ~ 0.8 nM (or 800 pmol/L) for G36A and GIP, ~1 nM for OXM, and ~10 nM (or ~ 350 pg/mL) for glucagon. All Ab-based analyses were carried out with either individual or pooled samples from 3–4 subjects and with three replicates of each. After the time-course incubation (0–96 hours), the plasma and serum samples were acidified with TFA to a final concentration of 0.1% to quench the proteolytic reactions and allowed for the ELISA buffer to neutralize that acid during processing, and frozen at -80°C until the next analysis.

For stability measurement in a whole blood sample, the targeted peptide was spiked into the fresh whole blood specimen, followed by a time-course incubation centrifugation. The rest of the procedures were the same as previously described for a plasma sample analysis. For testing the effect of Diprotin A (Sigma-Aldrich) on the stability of GLP-1, the inhibitor (500 unit/mL) was added into the collected whole blood sample prior to spiking the peptide, and followed by the above described procedures.

GLP-1 and GIP samples were supplied for Enzyme-Linked Immunosorbent Assay (ELISA) with the kits purchased from LINCO Research (St. Charles, MO), and the analyses were carried out according to the protocols suggested by the manufacturer. In GLP-1 analysis, 100 μL of the plasma sample quenched after time-course incubation was added into each microplate well, which had been loaded with 100 μL assay buffer provided with the kit. The microplate was incubated for 24 hours at 4°C, and followed by a standard ELISA protocol. Finally, the plate was read at an excitation/emission wavelength of 355 nm/460 nm in a plate reader purchased from Tecan US (Morrisville, NC). For GIP analysis, 20 μL of quenched samples was added into each well, and incubated for 1.5 hours at RT with gentle shaking, as per the protocols provided with the assay kit. The absorbance of each sample was measured at 450 nm and 590 nm in the plate reader.

OXM and glucagon measurements were performed by a competitive radioimmunoassay (RIA) with ^125^I-labeled OXM (Phoenix Pharmaceuticals, Inc., Burlingame, CA) and ^125^I-labeled glucagon (EMD Millipore, Billerica, MA) as the competitor, respectively. 300 μL of OXM sample, or 100 μL of glucagon sample was mixed with the primary antibody in a polystyrene tube and incubated overnight (22–24 hours) at 4°C, then mixed with the competitor and subjected to another overnight incubation at 4°C. The cold, precipitating reagent was added, vortexed, and incubated for 20 min at 4°C. The cpm (counts per minute) of the pellets was determined using a γ-counter (Beckman Coulter, Brea, CA).

### Digestion of OXM by DPP-IV

OXM was added into 100 μL of 50 mM HEPES buffer (pH7.5) up to 1 μM, followed by the addition of 1 unit of purified DPP-IV enzyme and incubation for time-dependent digestion at 37°C. At specified time periods, a 10 μL aliquot was withdrawn, desalted, and concentrated by Zip-Tip C18 (EMD Millipore, Billerica, MA); MALDI-TOF MS was used to monitor the cleavages of OXM by DPP-IV.

## Results and Discussion

With the advantage of high resolution and mass accuracy, MALDI-TOF MS enabled us to monitor both the parental peptide and its derived daughter peptides in a single spectrum [23]. We further measured the stability of metabolic peptides under *ex vivo* sampling conditions using both time-course MALDI-TOF MS analysis [36] and Ab-based immunoassays employed traditionally and extensively for peptide quantitation. Due to the high spiking concentration and C18 zipTip purification the endogenous peptides were suppressed in the MALDI spectrum and thus not interfering with our results.

### Instability of incretin in conventional plasma and serum samples

**Fig 1:**
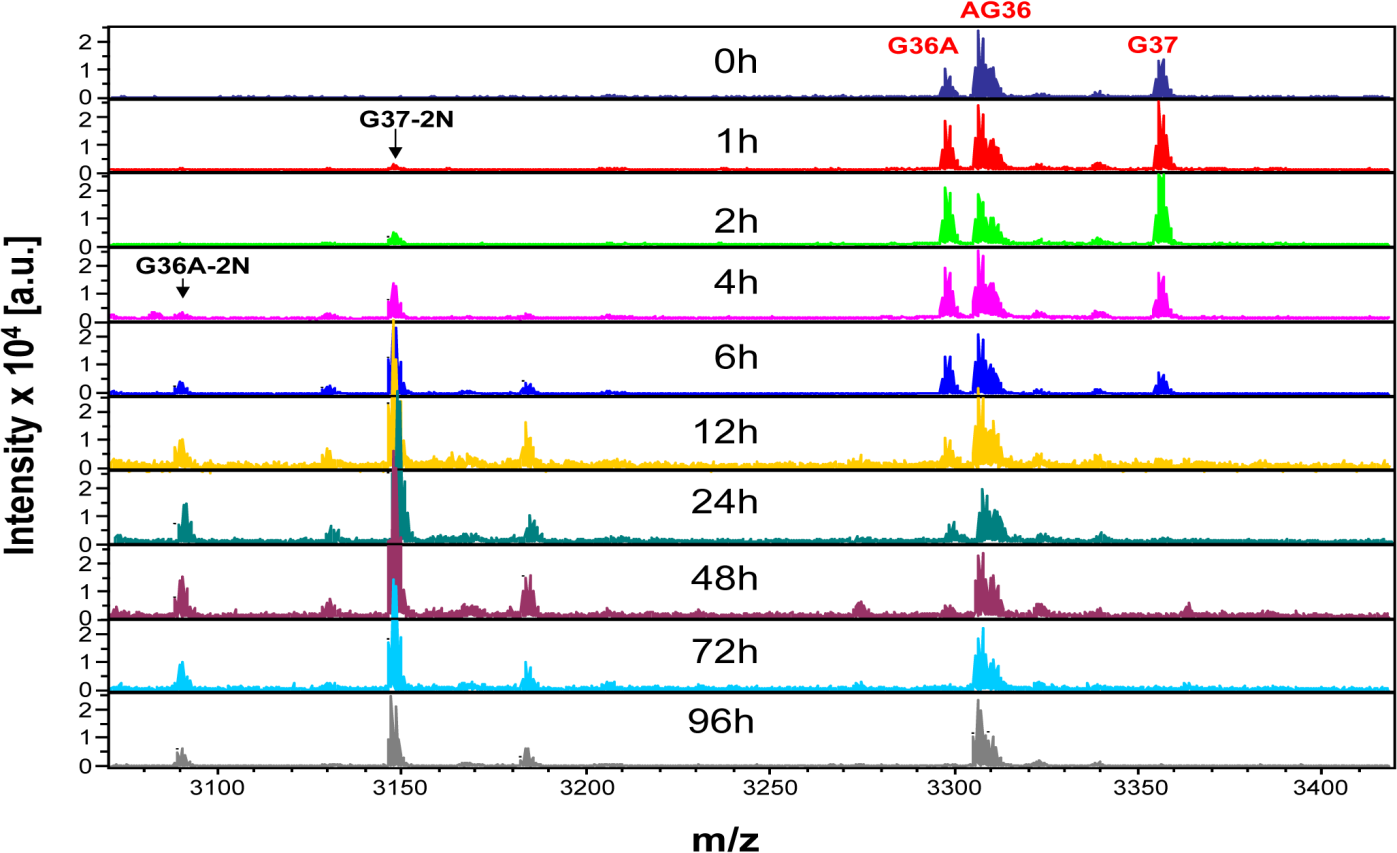
Instability of GLP-1 in EDTA plasma. Both active GLP-1 forms, G36A and G37, were spiked into EDTA plasma and incubated at room temperature. At indicated time points, the aliquots were withdrawn and AG36 was added into the plasma samples as control for relative quantitative analysis. While G36A and G37 decreased over incubation time, G36A-2N and G37-2N increased and then decreased. This is a typical figure from > 10 subjects; each subject has one or more replicate.

We first investigated the *ex vivo* stability of active GLP-1 and GIP in a widely used EDTA plasma sample by time-course MALDI-TOF MS to monitor peptide change during 4-day incubation at RT. As shown in Fig 1, the intensities of two active GLP-1 forms (G36A and G37), relative to the control peptide (AG36), decreased over time. Meanwhile, their two shorter fragments GLP-1 (9–37) (noted G37-2N, 3148.12 m/z) and GLP-1 (9-36A) (noted G36A-2N, 3088.06 m/z), both with two N-terminal residues removed (-2N), increased and then decreased, suggesting generation and degradation over time during the 4-day incubation (Fig 1). The generation of these -2N fragments clearly indicated that DPP-IV activity intrinsic to the plasma sample contributed to the removal of the two N-terminal residues of GLP-1; the degradation of these fragments implied that other proteolytic enzyme(s) also contributed to cleavages of the fragments and their parent peptides. Similar results were also observed when two active GLP-1 peptides were spiked into citrate- or heparin-plasma samples. Similar to the -2N cleavage of GLP-1 by DPP-IV, the -2N fragment (inactive GIP(3–42), 4749.65 m/z) of active GIP(1–42) was easily detected in EDTA plasma at two hours, and thereafter the fragment increased along with the decrease of its parental GIP(1–42) (S1A Fig). The same results were also observed when GIP (1–42) was incubated in a conventional serum sample (S1B Fig), suggesting similar DPP-IV activity in serum and plasma samples.

When two GLP-1 peptides, G36A and AG36, with the former peptide naturally amidated and the latter peptide synthesized without this modification, were simultaneously spiked into either a serum or plasma sample, the two peptides decreased over time (S2 Fig). Their daughter fragments, G36A-2N (at 3088.06 m/z), AG36-2N (at 3098.38 m/z as observed in EDTA plasma), and AG36-1C (at 3151.52 m/z) generated from the truncation of the C-terminal end residue of AG36, were detectable at the first 30 minutes and thereafter increased over time within six hours. The observation that AG36 decreased faster than G36A and AG36-1C (no G36A-1C) in the first 30 minutes (S2 Fig) demonstrated that the natural amidation on the C-terminal end of G36A prevented the peptide [37] from the -1C truncation by C-terminal exocarboxypeptidases. Most likely, the C-terminal truncation was by carboxypeptidase M (EC 3.4.16)[12], which removed specifically the C-terminal Arg residue [38] as in the AG36 sequence. However, we could not exclude the possibility that other exocarboxypeptidase(s) intrinsic to the plasma sample also contributed to this truncation. All of these results clearly indicated that both DPP-IV and peptidase (s) (at least exocarboxypeptidase M) contributed to the *ex vivo* instability of two active GLP-1 peptides, similar to the observation of the peptides in their *in vivo* metabolism [38]. The stability or half-life (t½) of both active GLP-1 forms in conventional plasma and serum samples were measured according to first-order kinetic degradation [23, 36] (Table 2). Clearly, both peptides have half-lives within only a few to several hours in these *ex vivo* traditional serum and plasma samples with EDTA, Citrate, or Heparin as an anticoagulant. Above all, the proteolysis-driven instability of the peptides may cause a large variation which prevents the accurate measurement of GLP-1 and GIP, and thus it is essential to preserve the peptides in *ex vivo* blood specimens when assessing their concentrations, such as, during incretin-based drug development.

**Table 2:** Stability (half-life) of peptide hormones in blood specimens.

Peptide	Sample	Temp^a^	t ½ (hrs) ^b^	Method
**(G36A**)	EDTA plasma	RT	4–24	MS, ELISA
		on ice	12 ± 3	ELISA
	Heparin Plasma	RT	13 ± 0.7	MS
	Citrate Plasma	RT	4 ± 0.2	MS
	Serum	RT	4 ± 0.2	MS
	P700 plasma	RT	>96	MS, ELISA
		on ice	>96	ELISA
	P800 plasma	RT	>96	MS, ELISA
		on ice	>96	ELISA
	EDTA whole blood	RT	2 ± 0.4	MS
		on ice	4–14	MS
	P800 whole blood	RT	10 ± 0.5	MS
		on ice	37–96	MS
**(G37)**	EDTA Plasma	RT	4–18	MS, ELISA
	P700 Plasma	RT	>96	MS, ELISA
	P800 Plasma	RT	>96	MS, ELISA
	EDTA Whole blood	RT	1 ± 0.3	MS
		on ice	5 ± 1.0	MS
	P800 Whole blood	RT	12 ± 1.0	MS
		on ice	41± 5.0	MS
**GIP(1–42)**	EDTA Plasma	RT	5–20	MS
	Serum	RT	5–20	MS
	P800 Plasma	RT	>96	MS
**OXM(1–37)**	EDTA Plasma	RT	< 24	MS
	P800 Plasma	RT	>72	MS
**Glucagon**	EDTA Plasma	RT	5–20	MS
	P800 Plasma	RT	45	MS

a RT representing 24 ±1°C, and on ice representing the sample kept either on ice or in temperature-controlled chamber at 4 ±1°C.

b A time range of half-life represents the measurements of multiple samples collected from different individuals, and mean ± STDEV representing the t½ measurement from a single sample with three replicates.

### Ex vivo stabilization of incretin in plasma with DPP-IV and enzyme inhibitors

**Fig 2:**
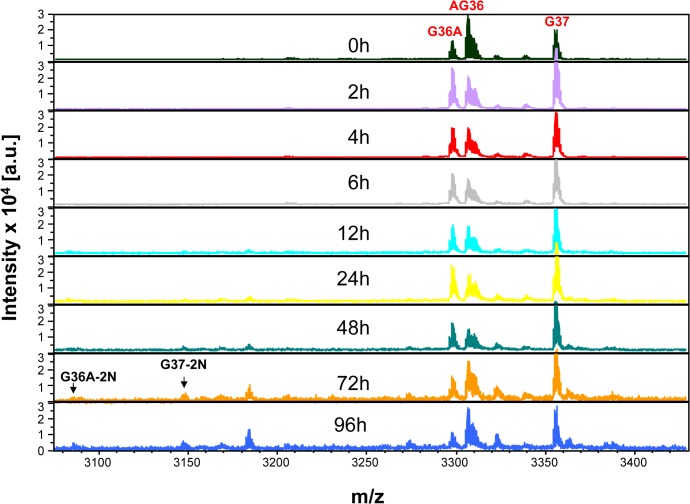
Stabilization of GLP-1 by enzymatic inhibitors. Both G36A and G37 were stable over 4-day incubation in P800 plasma, and their fragments, including G36A-2N and G37-2N, were only detectable after 72 hours. This is representative of 3 subjects.

To stabilize incretin and other gut peptide hormones (e.g. Glucagon and OXM), we screened, selected, and optimized a cocktail of enzymatic inhibitors that targeted proteases and peptidases, including DPP-IV. The inhibitor cocktail was included in evacuated blood collection tubes, BD P700 (for GLP-1preservation) and BD P800 (for GLP-1 and other metabolic peptides), to prevent the peptides from degradation during sample collection, processing, and transportation. Using the P800 or P700 plasma sample, we demonstrated that both active G36A and G37 peaks in the time-course MALDI-TOF MS were stabilized relative to the control (AG36) during 4-day incubation at RT (Fig 2). None of the shorter GLP-1 fragments was detected during the first 2-day incubation and only very small peaks of G37-2N and G36A-2N were detectable after 3-day incubation. These observations clearly demonstrated that the efficient inhibition of DPP-IV and other peptidase(s) activity was accomplished in the P800 (Fig 2) and P700 plasma samples.

**Fig 3:**
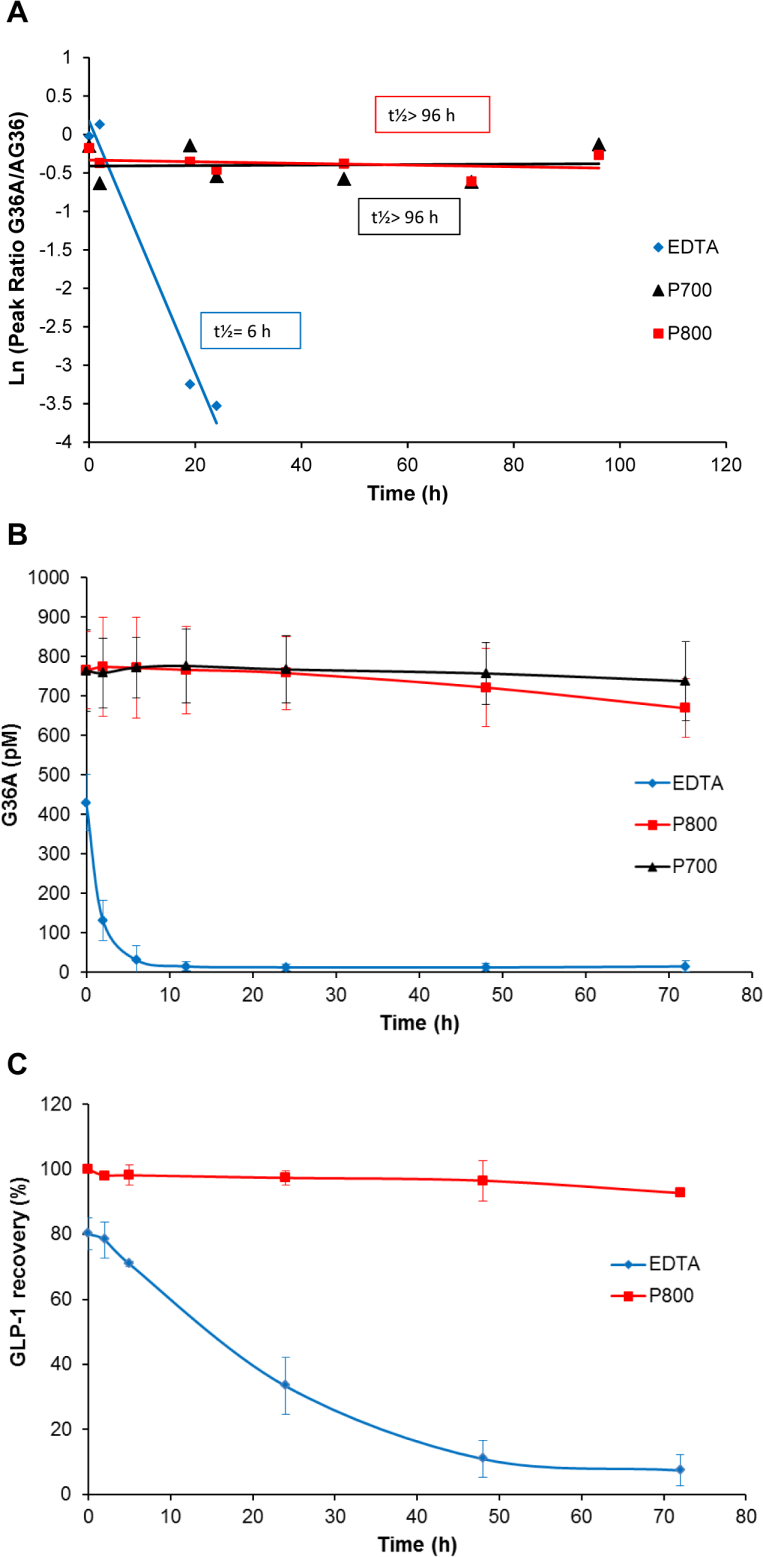
GLP-1 stabilization by Protease Inhibitors. **A. GLP-1 stability in EDTA, P700 and P800 plasma samples.** G36A was incubated in EDTA, P700 and P800 plasma samples prepared from the same three healthy individuals. The relative intensity (Rel. Int.) representing the ratios of peak intensity of G36A to AG36 was plotted according to the first-order degradation, and half-lives of the GLP-1 peptides were then determined. The recovery of the peptide is the ratio of the area of the peak for the peptides studied to the area of the control peptide; both peptides are spiked at the same final concentration. **B. GLP-1 stability comparison in EDTA and plasma samples with protease inhibitors at room temperature.** Four individual plasma samples collected with EDTA, P800, and P700 tubes were incubated with G36A and analyzed by ELISA. The analysis of G36A by ELISA was carried out with pooled plasma samples from 3 subjects with three replicates. The results displayed are representative. The error bars indicated standard deviations of the means from two replicates of four individuals. **C. GLP-1 stability in EDTA and P800 plasma samples on ice.** The analysis of G36A by ELISA was carried out with pooled plasma samples from 3–4 subjects with three replicates.

For a more quantitative comparison of the GLP-1 stability in P700, P800 and EDTA samples, the ratios of GLP-1 intensity to control (AG36) were plotted versus incubation time (Fig 3A), and the half-life of GLP-1 was determined [22]. The results further showed that the half-life (t½) of G36A in either P700 or P800 plasma samples was greater than 96 hours, while the same peptide in the blood specimen collected in the EDTA plasma tube had a half-life of 6.0 hours (Fig 3A). Similar results were also observed when G37 was tested in these samples (Fig 2 and Table 2). To obtain quantitative results, a G37 aqua peptide would have to be deployed. These results further demonstrated the stabilization of the two active GLP-1 peptides for more than four days (t½) by using the plasma samples with PIs (P700, P800).

The stability of active GLP-1 was further evaluated with an Ab-based method. With its advantage of high sensitivity (~ 1000 folder higher than MS in our experimental set up) and quantification, Ab-based immunoassay has been used extensively for protein and peptide quantifications. Using GLP-1 ELISA, we demonstrated that the concentration of active G36A remained without remarkable decrease within the first 72-hours in both P700 and P800 plasma samples at RT, while in EDTA plasma its concentration dropped quickly within the first five hours and was undetectable after 24 hours (Fig 3B). Even at “time 0,” when the samples were processed as quickly as possible (i.e. within 20 min) with no dwell time [34], approximately 50% of G36A was lost in the EDTA sample compared to that in the P800 or P700 sample at RT (Fig 3B). When a similar experiment was carried out in EDTA and P800 plasma samples on ice (~ 4°C), we also observed the time-dependent decrease of GLP-1 in EDTA with ~ 20% loss at “time 0”, and stabilization of the peptide in P800 over 72 hours (Fig 3C). This phenomenon was not observed in our MS results as the concentrations are higher (400 pM in Ab-based method and 0.4 μM in MS method). Although the degradation in the EDTA sample was slower at 4°C than at RT (comparison of Fig 3B and 3C), the estimated half-life of G36A on ice was still less than 15 hours. Furthermore, when G37 was used to replace G36A or when a P700 plasma sample was used to replace a P800 plasma sample, we obtained similar results of t½> 96 hours either on ice or at RT (Table 2). All of these results demonstrated that the degradation of active GLP-1 during plasma or serum sample collection and handling, specifically during the dwell time post-centrifugation [17, 32], was efficiently suppressed in both P700 and P800 plasma samples. However, this degradation could not be prevented by cooling of a conventional plasma or serum sample, further indicating the importance of including the efficient protease inhibitors for blood collection [22–24].

**Fig 4:**
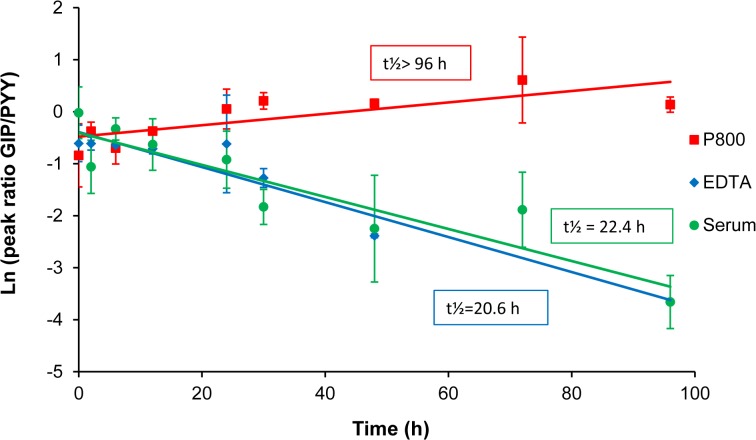
Stability of GIP in serum and plasma samples. Stability (T _½_) was indicated in the first-order kinetics analysis of GIP in pooled P800, EDTA and serum samples. The relative intensity (Rel. Int,) represented the peak ratio of GIP to the control (S1A, S1B and S1C Fig). Data are representative from 4 subjects with 1 or two replicates.

Time-course MALDI-TOF MS showed that GIP(1–42) was also stabilized in the P800 plasma sample with stable peak intensity (relative to control PYY) and without any GIP degradation fragments detectable during 4-day incubation at RT (S1C Fig). Its t½ was greater than 96 hours in the P800 plasma sample, significantly longer than its t½ of 20.6 and 22.4 hours in EDTA plasma and serum samples, respectively (Fig 4), in which active GIP(1–42) was turned into the inactive form GIP(3–42). Whether the P800 plasma sample was kept at RT or at 4°C and collected from an individual or pooled multiple individuals, the P800 samples always provided a t½ of active GIP> 96 hours. Unlike GLP-1 ELISA results, however, GIP time-course ELISA results did not reflect its instability in EDTA samples as current ELISA/RIA kits available on market could not selectively measure the active GIP(1–42) [39, 40]. Nevertheless, for active GIP assay development, it was suggested to use the P800 plasma sample [39].

### Stability of GLP-1 in whole blood samples

**Fig 5:**
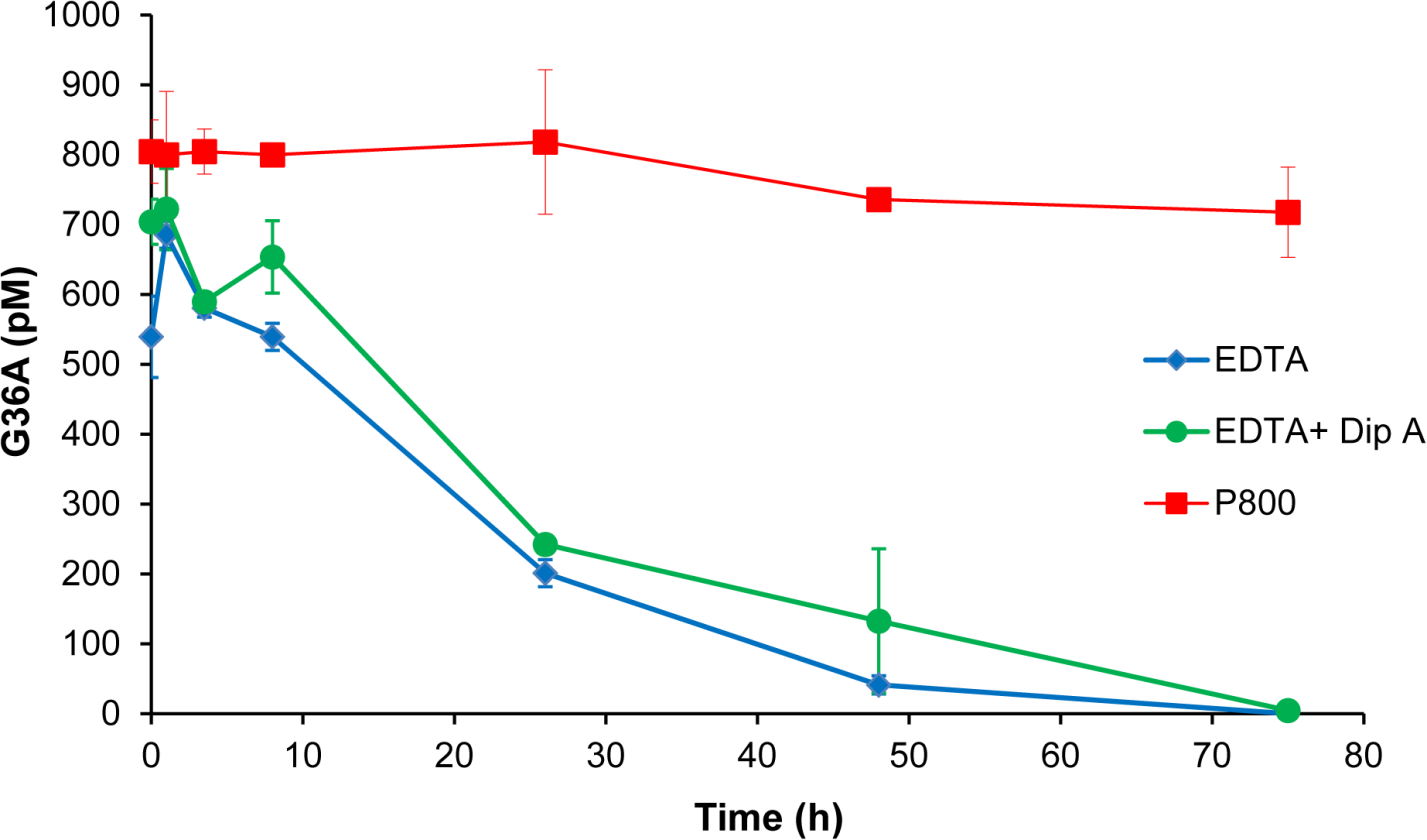
GLP-1 stability in whole blood. Active G36A was spiked into EDTA, EDTA plus Diprotin A (500 units/mL) and P800 whole blood samples. After incubation at room temperature for indicated time periods, the samples were centrifuged, and plasma portions were taken for ELISA-based peptide analysis. This data is representative of 1 subject with three replicates. A total of 3 subjects were tested and gave similar profiles.

In many blood-collection settings, the whole blood specimens may not be centrifuged and processed immediately (e.g., in many physicians’ offices where the blood specimens have to be transported to a centralized laboratory for processing and analysis). This dwell time of the specimens prior to centrifugation can be over 24 hours and cause significant variation of analytical results. To evaluate the stability of GLP-1 during this dwell time, we spiked GLP-1 peptides into pre-chilled (on ice or at 4°C chamber) P800 whole blood, EDTA whole blood, EDTA whole blood + Diprotin A (10μM, or 500 units/mL) samples. Diprotin A has previously been reported as a DPP-IV inhibitor [41–43], we included it in an EDTA whole blood sample and compared its protective effect with that of the P800 cocktail. Other therapeutic DPP-IV inhibitors may yield similar stabilization. The results indicated that G36A was stabilized in whole blood P800 samples with t½ greater than 72 hours, while its t½ in the EDTA whole blood samples was shorter than 20 hours and Diprotin A provided some stabilization of the peptide (t½ ~ 20 hours) (Fig 5). Our observation of Diprotin A was consistent with a recent report that Diprotin A (0.5 mM) did not induce resistance in GLP-1 cleavage in mouse and human Hepatocytes [43], but was inconsistent with the early report that Diprotin A (0.1 mM) could fully suppress the -2N cleavage of GLP-1 by DPP-IV in human serum [31]. This inconsistency could be explained by the difference in the sample types: a whole blood sample vs. a serum sample (see below). When stability experiments were carried out in whole blood specimens at RT, the measured t½ of G36A and G37 was 9.9, and 12.5 hours respectively in P800, and only 1.3 and 1.9 hours respectively in an EDTA whole blood sample (S3 Fig and Table 2), again showing the benefit of the P800 sample.

The two active GLP-1’s, as well as the other peptides tested, had longer half-lives in *ex vivo* whole blood samples (Table 2) than in those reported in *in vivo* circulation [3, 29]. This difference can be explained by at least three reasons: (i) temperature difference with RT (24 ± 2°C) in *ex vivo* samples vs. 37°C in circulation, which is more favorable to protease reactivity; (ii) EDTA included in an *ex vivo* blood specimen as an anticoagulant is also an effective inhibitor to suppress cation-dependent proteases (e.g. Matrix metalloproteinases), and thus contributes to the stability of *ex vivo* proteins and peptides [22, 44]; (iii) there are extra DPP-IV activities in *in vivo* circulation than in *ex vivo* samples. In fact, DPP-IV is also expressed on the surface of endothelial cells [38], which have multiple biological functions, including critical basal and inducible metabolic functions [45].

By comparison of plasma and whole blood results, we also observed that two active GLP-1 peptides showed higher instability in a whole blood sample than in a separated plasma sample. As an active tissue, the *ex vivo* whole blood sample retains its metabolic activities with many proteolytic enzymes located on cell surfaces. In addition to its soluble form of DPP-IV in blood plasma, the enzyme is also localized on leukocyte subsets (activated T cells, natural killer cells, B cells) and mature thymocytes in whole blood [38]. Thus, we expect extra DPP-IV activity in a whole blood sample as compared to a plasma sample. It is also possible that DPP-IV on a cell surface has different specificity than its soluble form. These differences suggest that it is more challenging to stabilize a targeted peptide biomarker in a whole blood sample than in a separated plasma sample, and may also provide an explanation as to why Diprotin A conveys little protection to GLP-1 in whole blood (Fig 5), but is effective in inhibiting DPP-IV activity in human serum [41]. Therefore, separation of the plasma portion from whole blood as soon as possible after blood is collected, is suggested to minimize the proteolytic variability [34] and is also recommended for GLP-1 measurement.

It is also well known that the protease activities are slowed down when the storage temperature is lower. However, keeping a whole blood sample at 4°C and even adding Diprotin A to the samples do not effectively suppress the fast degradation of GLP-1. Furthermore, platelets are activated and hemolysis (red blood cell lysis) increases at 4°C, both negatively impacting the quality of blood samples. Therefore, we do not suggest utilizing whole blood at 4°C in general clinical research applications.

Importantly, both active GLP-1 forms are highly stabilized in P800 and P700 samples with t½ > 96 and > 9.9 hours in plasma and whole blood samples at RT, respectively. Both peptides are further stabilized in the whole blood P800 sample at 4°C with t½ >72 hours. Therefore, we suggest using P800 or P700 tubes for blood collection for GLP-1 measurement as well as centrifuging and separating the plasma from blood cells as soon as possible or within 30 minutes. However, keeping the whole blood sample at 4°C rather than at RT is a better choice for GLP-1 stabilization if centrifugation is not possible within 30 minutes.

### Degradation and Stabilization of OXM

**Fig 6:**
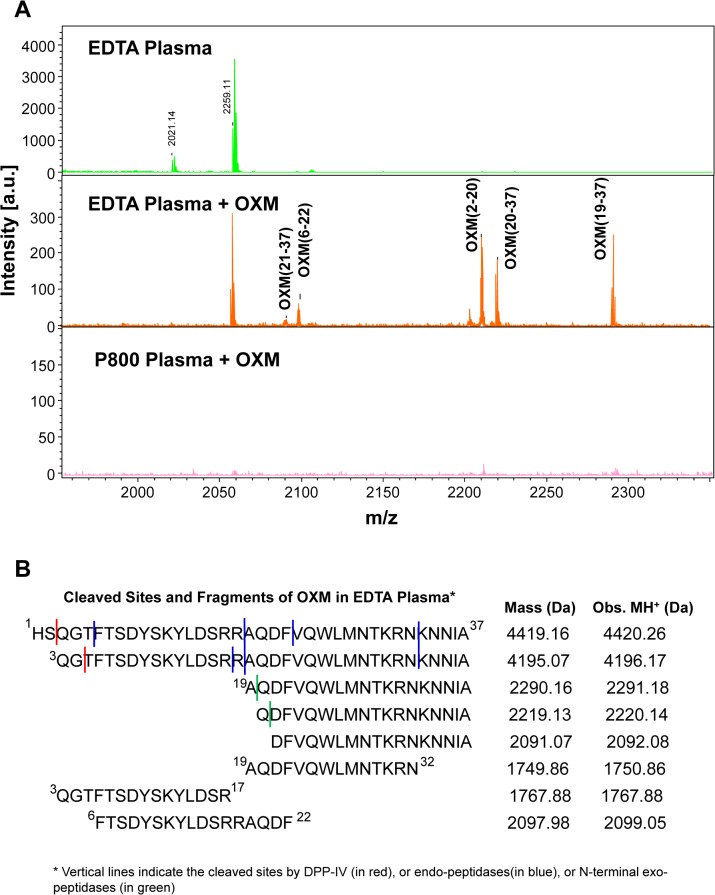
OXM fragments detected during the incubation of full-length OXM(1–37) in plasma sample. **A-** Typical peptide spectra at 24-hour incubation of the spiked full-length OXM-K^33^ in plasma samples from 3 subjects repeated in duplicates, **B**- summary of OXM fragment peptides observed during the first 48-hour incubation of EDTA plasma spiked with OXM(1–37).

The degradations of OXM and generations of its daughter peptides over time were monitored by MS during the incubations of an EDTA plasma sample, a P800 plasma sample spiked with full length OXM (1–37), and a non-spiked EDTA plasma sample as a control. Endogenous fl OXM could not be detected in EDTA plasma by MALDI-MS; therefore unspiked P800 plasma was not included. The spectra showed that extra peptides were detected in the EDTA sample spiked with OXM(1–37) compared with the non-spiked EDTA and spiked P800 samples (Fig 6A). Most of these additional peptides detected during the first 48 hours of incubation were identified as OXM fragments generated from the proteolytic degradation of the spiked OXM (1–37) (Fig 6A and 6B), including OXM-2N (S4A Fig).

**Fig 7:**
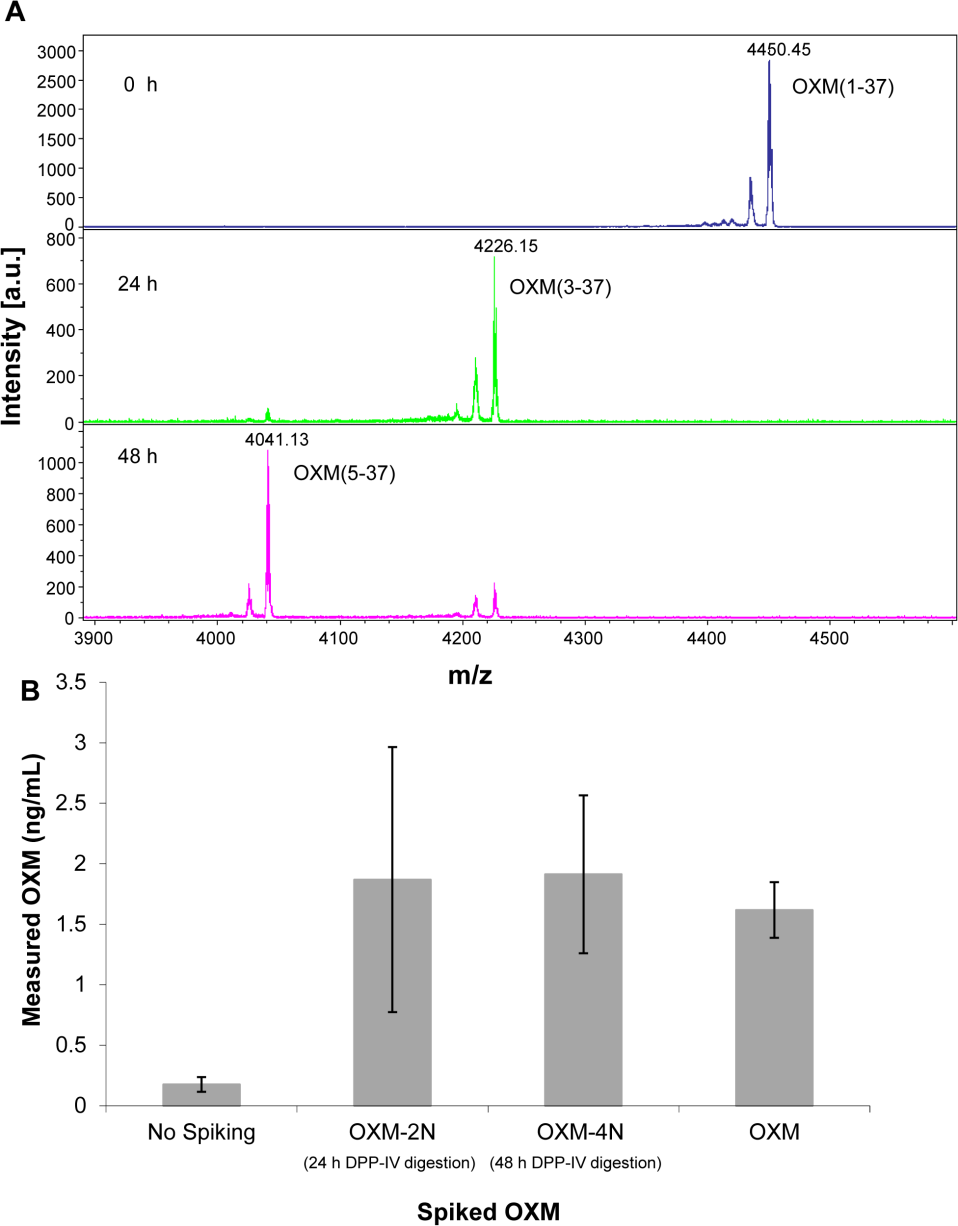
A. Digestion of OXM by purified DPP-IV enzyme. OXM(1–37) was incubated with DPP-IV in 100 mM HEPES buffer for 0, 24, 48 hours. The peptides were analyzed using MALDI-TOF MS. B. Measurements of OXM fragments by EIA. Full-length OXM (1–37), fragments OXM-2N and OXM-4N generated by incubation with pure DPP-IV for 24 and 48 h respectively, were spiked into P800 plasma, immediately acidified, and analyzed by EIA. The data are from 3 subjects with 2 replicates.

The generation of OXM-2N fragment from fl OXM in EDTA sample also resulted from DPP-IV cleavage [31, 32, 46], which was confirmed by the time-course MS analysis of incubation of fl OXM(1–37) with purified DPP-IV enzyme. This experiment was performed to identify digestion products from the fl OXM by DPP-IV which is why we chose to perform the digestion in HEPES rather than the physiological plasma. The results demonstrated that DPP-IV turned OXM(1–37) (at 4450.45 m/z) into OXM-2N (OXM(3–37) (at 4226.15 m/z) in the first 24 hours, and further turned the generated OXM-2N into OXM-4N OXM(5–37) (at 4041.13 m/z) (Fig 7A). Not surprisingly, it was the first time we observed that the DPP-IV enzyme could further remove the next two residues (QG) on the N-terminal end of OXM-2N, as DPP-IV had specificity to Gly (G) residue in the penultimate position when the first residue (Gln in this case) was neutral [41]. This sequential two-step -2N reaction of DPP-IV was also observed on the cleavages of vasoactive intestinal peptide (VIP), pituitary adenylate cyclase-activating peptide (PACAP27 and PACAP38), and gastrin-releasing peptide (GRP) [46].

Other detected OXM fragments in the spiked EDTA plasma sample were expected to be a result of other proteolytic activities intrinsic to the EDTA plasma sample. Specifically, peaks at 2291.18, 2220.14, and 2092.08 m/z were mapped to OXM(19–37), OXM(20–37), and OXM(21–37) (Fig 6A and 6B), respectively. Mechanistically, a trypsin-like endopeptidase cleaved on the C-terminal side of Arg^18^ residue of OXM(1–37) or OXM(3–37) to release OXM(19–37), and the generated peptide OXM(19–37) was truncated by exopeptidase(s) from its N-terminal end of the peptide into OXM(20–37), which was further truncated into OXM(21–37) (Fig 6A and 6B). This typical process described as a Sequential Multi-Step Reaction (SMSR) in a previous report [23] was a typical protein and peptide degradation observed in *ex vivo* plasma samples [22], and should be also applicable to the kinetic metabolism of proteins and peptide in *ex vivo* circulation. Furthermore, not-yet defined peptidases also contribute to the instability by nonspecifically cleaving at the C-terminal sides of T^5^, F^22^, and N^32^, which led to the generations of OXM(6–22) and OXM(19–32) (Fig 6A and 6B). These detected fragments clearly demonstrated that both DPP-IV (S4A Fig) and other endopeptidase(s) and exopeptidase(s) [22, 28] contributed to the degradation of OXM in the EDTA plasma sample. As OXM has become an attractive therapeutic agent, these cleavable sites mapped by MS analysis (Fig 6B) enable the targeted amino acid residues to be modified for designing long-lasting OXM analogs against obesity and diabetic diseases.

Importantly, all OXM fragments detected in the spiked EDTA sample were not detectable during 3-day incubation of the spiked P800 sample. Meanwhile, the full-length OXM was stabilized for more than three days (S5 Fig), and four days in time-course EIA analysis (S5 Fig). Two other peptides, 2021.14 and 2259.11 m/z, detected in non-spiked or spiked EDTA samples were also not detectable in P800 samples (Fig 6A). The former peptide was identified as complement C3f (mass: 2020.10 Da) from proteolytic digestion of complement component 3 [22, 47]; the latter peptide was not-yet identified but was expected to be also resulted from the cleavages of endogenous plasma proteins or larger peptides by proteolytic enzymes. All of these observations further demonstrated that, in addition of DPP-IV, other protease(s) and/or peptidase(s) intrinsic to the traditional EDTA plasma sample were also inhibited in P800 plasma samples.

However, the time-course EIA result did not reflect the instability of fl OXM in the EDTA sample (S5 Fig).To further test if the Ab used in this EIA analysis kit recognized the N-terminal residues of OXM, we digested the fl OXM with purified DPP-IV for 24 and 48 hours to generate OXM-2N and OXM-4N, respectively (Fig 7A), spiked the same amount of each of these peptides into the same P800 samples, and followed with EIA analysis. The results showed no remarkable difference in OXM measurements among these three peptides (Fig 7B), and demonstrated that the immunoassay for OXM did not distinguish these three peptides, while it provided a high sensitivity for the total peptide quantitation.

### Stability of glucagon in plasma

Similar investigation with time-course MS and RIA methods was also carried out for the stability of glucagon, another peptide substrate of DPP-IV enzyme [30, 31, 33]. Although its stability measured by MS analysis showed a larger individual-to-individual variability in EDTA samples with t½ spanning from 5 to 24 hours, its half-life in P800 samples was consistently greater than 45 hours (Table 2, S6A Fig). Similar to Ab-based results of OXM and GIP, the time-course RIA results showed no significant difference between EDTA and P800 samples (S6B Fig), suggesting that this Ab-based assay did not reflect the instability of glucagon.

Traditional Ab-based immunoassay has been widely used for protein and peptide quantification. While the immunoassay provides a high sensitivity and high through-put, both of which are necessary in current clinical applications, its specificity has to be characterized or confirmed before it is selected for the quantitative analysis. Among four immunoassays used for detection of active peptides in this study, only one (for GLP-1) can recognize the active peptide, and the other three (GIP, OXM and glucagon) could not distinguish the full length or active peptides from their proteolytic fragments, which caused disparity between Ab-based and MS-based results. However, once the Ab-based assay is specific to the targeted peptide (e.g. active GLP-1), its stability results are consistent with those obtained by MS. It is important to note that the range of concentrations used in spiking for MS experiments were unphysiological and that caution should always be used when comparing results from different concentrations and methods of detection. It is feasible to develop a specific Ab-based assay for active GIP(1–42) measurement by characterizing and selecting specific monoclonal Abs which can recognize the two N-terminal residues [39]. However, it will be very challenging to develop highly specific Abs to recognize only OXM or only glucagon, since the N-terminal 29 A.A. residues of OXM share the entire sequence as glucagon (Table 1) and glicentin (with 69 A.A.) contains the entire molecules of OXM(1–37) with extended 32 A.A. residues in its N-terminal side [48, 49].

However, MS-based analysis with its high specificity can easily detect and distinguish the targeted peptides from their fragments. As Mass Spectrometry continues to increase sensitivity with methods such as LC MS/MS SRM (single reaction monitoring) the quantification of proteins and peptides are likely to make this approach more feasible for a wider range of low abundant peptides [50–52], including GLP-1 [53], OXM [54], GIP [55, 56], and glucagon [57]. When capabilities are available, preference should be given to MS methods allowing measurement of native peptide hormones such as single reaction monitoring (SRM). With continuous reduction of its cost and increase in its sensitivity, the MS-based method is currently attracting more attentions for development in analytical biopharmaceuticals [51] and in clinical applications [57]. Therefore, to investigate peptide stability, we recommend using the MS-based method for specific peptide quantification or for characterizing the active peptide and its fragments measured by an Ab-based assay before selection for quantitation. A better appreciation for the biological significance between active peptide and its fragments and distinguishing between the two by either MS-based or Ab-based method would play an important role in peptide-based drug and biomarker development in the near future.

Nevertheless, our results from time-course analyses of GLP-1, GIP, OXM, and glucagon indicated their instability in conventional blood specimens. We showed the stabilization of these variable peptides by selecting the inhibited samples, and demonstrated that the P800 blood collection tube provided a robust blood specimen to measure the active four peptide hormones.

## Conclusions

Our investigations on the preanalytical variability of blood specimens have reiterated that proteolytic degradation intrinsic to blood specimens causes preanalytical instability of plasma peptides and the stabilization of peptide drug or biomarker candidates is required for their accurate measurements. Specifically, we demonstrated the proteolytic instability of four gut hormone peptides, including GLP-1, GIP, Glucagon, and OXM, in *ex vivo* blood specimens. DPP-IV activity contributed to their instability by removing the N-terminal two residues of these four peptide hormones, and it also further removed the next two N-terminal residues of OXM(3–37) to form OXM(5–37). For this work we estimate the concentration of DPP-IV was approximately 1.03 μg/mL (56). Future studies could investigate the correlation between DPP-IV concentration and DPP-IV peptide substrate stability. Other endopeptidase and exopeptidase activities also played roles in cleavages of OXM in multiple sites, and carboxypeptide M activity contributed to the digestion of GLP-1. These proteolytic degradations were mechanistically either SMSR or non-specific cleavages. Importantly, all of these proteolytic-enzyme-driven degradations of GLP-1, GIP, OXM and glucagon were efficiently inhibited in P800 plasma samples, and thus P800 ensured their *ex vivo* stabilization with t½ > 96, 96, 72, and 45 hours at RT, respectively. P700 plasma also stabilized GLP-1 with t½ > 96 hours (Table 2). Such stabilization should enable accurate measurements of these gut peptide hormones and/or their analogs for drug and/or biomarker development as well as allow to reduce the significance of other preanalytical source of errors.

## Supporting Information

S1 Fig Time-course MALDI-TOF MS of GIP in *ex vivo* plasma and serum samples. The peaks of GIP(1–42) and GIP(3–42) were indicated. The decrease of GIP(1–42) and increase of GIP(3–42) over incubation periods of time demonstrated that DPP-IV activity contributed to the degradation of GIP in the EDTA plasma (A) and serum (B) samples. The stable peak of GIP(1–42) was observed in the P800 plasma sample at room temperature. (C) Data are representative of 4 subjects with 1 or 2 replicates.
(TIF)

S2 Fig Digestion of GLP-1 by DPP-IV and carboxypetidase activities. G36A and AG36 were spiked into and mixed with serum sample in 1:10 ratio (v/v). The sample was incubated at room temperature and aliquots were withdrawn for peptide extraction and MALD-TOF MS analysis at indicated time points. Both daughter peptides G36A-2N and G36-1C were detected after incubation for 30 minutes. Data are representative from 3 subjects tested in duplicate.
(TIF)

S3 Fig Active GLP-1 stability in whole blood specimens. Two active GLP-1 forms (G36A and G37) were spiked into blood specimens freshly collected in EDTA and P800 tubes. The blood samples were incubated at room temperature for specified periods of time, centrifuged to collect plasma samples, followed by spiking AG36 as the internal control, and processed for MALDI-TOF MS analysis. The relative peak areas were plotted vs. the incubation time, and t½ of two peptides was determined. Data are from 3 subjects processed in duplicates.
(TIF)

S4 Fig OXM stability comparison between EDTA and P800 plasma samples at room temperature. Time-course MS of OXM in EDTA plasma sample (A)EDTA plasma indicates its instability with the generation of OXM-2N. (B) P800 Plasma The preservation of OXM (1–37) was achieved for up to 72 hours. The data are representative from at least 6 subjects.
(TIF)

S5 Fig OXM stability analyzed by EIA. Time course was performed.
(TIF)

S6 Fig Glucagon stability in EDTA and P800 plasma samples. (A)Time-course MS (B) Time-course EIA. Data are from 1 subject in triplicate.
(TIF)

## References

[1] Holst J, Ørskov C. In: Walsh J, Dockray G, editors. Gut peptides: biochemistry and physiology New York: Raven Press; 1994 p. 305–40.

[2] Stanley S, Wynne K, McGowan B, Bloom S. Hormonal regulation of food intake. Physiol Rev. 2005;85(4):1131–58. Epub 2005/09/27. 85/4/1131 [pii] 10.1152/physrev.00015.2004 . 16183909

[3] Baggio LL, Drucker DJ. Biology of incretins: GLP-1 and GIP. Gastroenterology. 2007;132(6):2131–57. Epub 2007/05/15. S0016-5085(07)00580-X [pii] 10.1053/j.gastro.2007.03.054 . 17498508

[4] Holst JJ, Vilsboll T, Deacon CF. The incretin system and its role in type 2 diabetes mellitus. Mol Cell Endocrinol. 2009;297(1–2):127–36. Epub 2008/09/13. 10.1016/j.mce.2008.08.012 S0303-7207(08)00362-6 [pii]. . 18786605

[5] Kim W, Egan JM. The role of incretins in glucose homeostasis and diabetes treatment. Pharmacol Rev. 2008;60(4):470–512. Epub 2008/12/17. 10.1124/pr.108.000604 pr.108.000604 [pii]. 19074620 PMC2696340

[6] Gautier JF, Fetita S, Sobngwi E, Salaun-Martin C. Biological actions of the incretins GIP and GLP-1 and therapeutic perspectives in patients with type 2 diabetes. Diabetes Metab. 2005;31(3 Pt 1):233–42. Epub 2005/09/06. MDOI-DM-06-2005-31-3-1262-3636-101019-200514505 [pii]. . 16142014 10.1016/s1262-3636(07)70190-8

[7] Nauck M, Smith U. Incretin-based therapy: how do incretin mimetics and DPP-4 inhibitors fit into treatment algorithms for type 2 diabetic patients? Best Pract Res Clin Endocrinol Metab. 2009;23(4):513–23. Epub 2009/09/15. 10.1016/j.beem.2009.03.002 S1521-690X(09)00021-9 [pii]. . 19748068

[8] Garber AJ. Incretin therapy—present and future. Rev Diabet Stud. 2011;8(3):307–22. Epub 2012/01/21. 10.1900/RDS.2011.8.307 22262069 PMC3280666

[9] Skibicka KP. The central GLP-1: implications for food and drug reward. Front Neurosci. 2013;7:181 Epub 2013/10/18. 10.3389/fnins.2013.00181 24133407 PMC3796262

[10] Maida A, Lovshin JA, Baggio LL, Drucker DJ. The glucagon-like peptide-1 receptor agonist oxyntomodulin enhances beta-cell function but does not inhibit gastric emptying in mice. Endocrinology. 2008;149(11):5670–8. Epub 2008/08/02. doi: 10.1210/en.2008-0336 en.2008-0336 [pii]. . 18669601 10.1210/en.2008-0336

[11] Cohen MA, Ellis SM, Le Roux CW, Batterham RL, Park A, Patterson M, et al Oxyntomodulin suppresses appetite and reduces food intake in humans. J Clin Endocrinol Metab. 2003;88(10):4696–701. Epub 2003/10/15. 10.1210/jc.2003-030421 . 14557443

[12] Bhat VK, Kerr BD, Flatt PR, Gault VA. A novel GIP-oxyntomodulin hybrid peptide acting through GIP, glucagon and GLP-1 receptors exhibits weight reducing and anti-diabetic properties. Biochem Pharmacol. 2013;85(11):1655–62. Epub 2013/03/23. 10.1016/j.bcp.2013.03.009 S0006-2952(13)00190-1 [pii]. . 23518155

[13] Ali S, Drucker DJ. Benefits and limitations of reducing glucagon action for the treatment of type 2 diabetes. Am J Physiol Endocrinol Metab. 2009;296(3):E415–21. Epub 2009/01/01. doi: 10.1152/ajpendo.90887.2008 90887.2008 [pii]. . 19116373 10.1152/ajpendo.90887.2008

[14] White CM. A review of potential cardiovascular uses of intravenous glucagon administration. J Clin Pharmacol. 1999;39(5):442–7. Epub 1999/05/11. . 10234590

[15] Kolb A, Rieder S, Born D, Giese NA, Giese T, Rudofsky G, et al Glucagon/insulin ratio as a potential biomarker for pancreatic cancer in patients with new-onset diabetes mellitus. Cancer Biol Ther. 2009;8(16):1527–33. Epub 2009/07/03. 9006 [pii]. . 19571666 10.4161/cbt.8.16.9006

[16] Hewage CM, Venneti KC. Structural aspects of gut peptides with therapeutic potential for type 2 diabetes. ChemMedChem. 2013;8(4):560–7. Epub 2013/01/08. 10.1002/cmdc.201200445 . 23292985

[17] Greco V, Pieragostino D, Piras C, Aebersold R, Wiltfang J, Caltagirone C, et al Direct analytical sample quality assessment for biomarker investigation: qualifying cerebrospinal fluid samples. Proteomics. 2014;14(17–18):1954–62. 10.1002/pmic.201300565 . 25044759

[18] Pieragostino D, Petrucci F, Del Boccio P, Mantini D, Lugaresi A, Tiberio S, et al Pre-analytical factors in clinical proteomics investigations: impact of ex vivo protein modifications for multiple sclerosis biomarker discovery. Journal of proteomics. 2010;73(3):579–92. 10.1016/j.jprot.2009.07.014 . 19666151

[19] Rai AJ, Gelfand CA, Haywood BC, Warunek DJ, Yi J, Schuchard MD, et al HUPO Plasma Proteome Project specimen collection and handling: towards the standardization of parameters for plasma proteome samples. Proteomics. 2005;5(13):3262–77. Epub 2005/07/30. 10.1002/pmic.200401245 . 16052621

[20] Rai AJ, Vitzthum F. Effects of preanalytical variables on peptide and protein measurements in human serum and plasma: implications for clinical proteomics. Expert Rev Proteomics. 2006;3(4):409–26. Epub 2006/08/12. 10.1586/14789450.3.4.409 . 16901200

[21] Percy AJ, Parker CE, Borchers CH. Pre-analytical and analytical variability in absolute quantitative MRM-based plasma proteomic studies. Bioanalysis. 2013;5(22):2837–56. Epub 2013/11/22. 10.4155/bio.13.245 . 24256362

[22] Yi J, Kim C, Gelfand CA. Inhibition of intrinsic proteolytic activities moderates preanalytical variability and instability of human plasma. J Proteome Res. 2007;6(5):1768–81. Epub 2007/04/07. 10.1021/pr060550h . 17411080

[23] Yi J, Liu Z, Craft D, O'Mullan P, Ju G, Gelfand CA. Intrinsic peptidase activity causes a sequential multi-step reaction (SMSR) in digestion of human plasma peptides. J Proteome Res. 2008;7(12):5112–8. Epub 2009/04/16. 10.1021/pr800396c . 19367699

[24] Craft D, Yi J, Gelfand CA. Time-Dependent and Sample-to-Sample Variations in Human Plasma Peptidome are Both Minimized Through Use of Protease Inhibitors. Analytical Letters. 2009;42(10):1398–406.

[25] Findeisen P, Sismanidis D, Riedl M, Costina V, Neumaier M. Preanalytical impact of sample handling on proteome profiling experiments with matrix-assisted laser desorption/ionization time-of-flight mass spectrometry. Clin Chem. 2005;51(12):2409–11. Epub 2005/11/25. 51/12/2409 [pii] 10.1373/clinchem.2005.054585 . 16306114

[26] Karsan A, Eigl BJ, Flibotte S, Gelmon K, Switzer P, Hassell P, et al Analytical and preanalytical biases in serum proteomic pattern analysis for breast cancer diagnosis. Clin Chem. 2005;51(8):1525–8. Epub 2005/06/14. clinchem.2005.050708 [pii] 10.1373/clinchem.2005.050708 . 15951319

[27] McLerran D, Grizzle WE, Feng Z, Bigbee WL, Banez LL, Cazares LH, et al Analytical validation of serum proteomic profiling for diagnosis of prostate cancer: sources of sample bias. Clin Chem. 2008;54(1):44–52. Epub 2007/11/06. clinchem.2007.091470 [pii] 10.1373/clinchem.2007.091470 17981926 PMC3354530

[28] Bystrom CE, Salameh W, Reitz R, Clarke NJ. Plasma renin activity by LC-MS/MS: development of a prototypical clinical assay reveals a subpopulation of human plasma samples with substantial peptidase activity. Clin Chem. 2010;56(10):1561–9. Epub 2010/08/27. 10.1373/clinchem.2010.146449 clinchem.2010.146449 [pii]. . 20739638

[29] Deacon CF. Circulation and degradation of GIP and GLP-1. Horm Metab Res. 2004;36(11–12):761–5. Epub 2005/01/19. 10.1055/s-2004-826160 . 15655705

[30] Zhu L, Tamvakopoulos C, Xie D, Dragovic J, Shen X, Fenyk-Melody JE, et al The role of dipeptidyl peptidase IV in the cleavage of glucagon family peptides: in vivo metabolism of pituitary adenylate cyclase activating polypeptide-(1–38). J Biol Chem. 2003;278(25):22418–23. Epub 2003/04/12. 10.1074/jbc.M212355200 M212355200 [pii]. . 12690116

[31] Mentlein R. Dipeptidyl-peptidase IV (CD26)—role in the inactivation of regulatory peptides. Regul Pept. 1999;85(1):9–24. Epub 1999/12/10. S0167-0115(99)00089-0 [pii]. . 10588446 10.1016/s0167-0115(99)00089-0

[32] Kervran A, Dubrasquet M, Blache P, Martinez J, Bataille D. Metabolic clearance rates of oxyntomodulin and glucagon in the rat: contribution of the kidney. Regul Pept. 1990;31(1):41–52. Epub 1990/10/29. 0167-0115(90)90194-2 [pii]. . 2270317 10.1016/0167-0115(90)90194-2

[33] Hinke SA, Pospisilik JA, Demuth HU, Mannhart S, Kuhn-Wache K, Hoffmann T, et al Dipeptidyl peptidase IV (DPIV/CD26) degradation of glucagon. Characterization of glucagon degradation products and DPIV-resistant analogs. J Biol Chem. 2000;275(6):3827–34. Epub 2000/02/08. . 10660533 10.1074/jbc.275.6.3827

[34] Yi J, Craft D, Gelfand CA. Minimizing preanalytical variation of plasma samples by proper blood collection and handling. Methods Mol Biol. 2011;728:137–49. Epub 2011/04/07. 10.1007/978-1-61779-068-3_8 . 21468945

[35] Carles-Bonnet C, Martinez J, Jarrousse C, Aumelas A, Niel H, Bataille D. H-Lys-Arg-Asn-Lys-Asn-Asn-OH is the minimal active structure of oxyntomodulin. Peptides. 1996;17(3):557–61. Epub 1996/01/01. 0196-9781(96)00001-0 [pii]. . 8735987 10.1016/0196-9781(96)00001-0

[36] Yi J, Liu Z, Gelfand CA, Craft D. Investigation of peptide biomarker stability in plasma samples using time-course MS analysis. Methods Mol Biol. 2011;728:161–75. Epub 2011/04/07. 10.1007/978-1-61779-068-3_10 . 21468947

[37] Wettergren A, Pridal L, Wojdemann M, Holst JJ. Amidated and non-amidated glucagon-like peptide-1 (GLP-1): non-pancreatic effects (cephalic phase acid secretion) and stability in plasma in humans. Regul Pept. 1998;77(1–3):83–7. Epub 1998/11/11. S0167-0115(98)00044-5 [pii]. . 9809800 10.1016/s0167-0115(98)00044-5

[38] Mentlein R. Mechanisms underlying the rapid degradation and elimination of the incretin hormones GLP-1 and GIP. Best Pract Res Clin Endocrinol Metab. 2009;23(4):443–52. Epub 2009/09/15. 10.1016/j.beem.2009.03.005 S1521-690X(09)00024-4 [pii]. . 19748062

[39] Troutt JS, Siegel RW, Chen J, Sloan JH, Deeg MA, Cao G, et al Dual-monoclonal, sandwich immunoassay specific for glucose-dependent insulinotropic peptide1-42, the active form of the incretin hormone. Clin Chem. 2011;57(6):849–55. Epub 2011/04/26. 10.1373/clinchem.2010.159954 clinchem.2010.159954 [pii]. . 21515744

[40] Deacon CF, Nauck MA, Meier J, Hucking K, Holst JJ. Degradation of endogenous and exogenous gastric inhibitory polypeptide in healthy and in type 2 diabetic subjects as revealed using a new assay for the intact peptide. J Clin Endocrinol Metab. 2000;85(10):3575–81. Epub 2000/11/04. 10.1210/jcem.85.10.6855 . 11061504

[41] Mentlein R, Gallwitz B, Schmidt WE. Dipeptidyl-peptidase IV hydrolyses gastric inhibitory polypeptide, glucagon-like peptide-1(7–36)amide, peptide histidine methionine and is responsible for their degradation in human serum. Eur J Biochem. 1993;214(3):829–35. Epub 1993/06/15. . 8100523 10.1111/j.1432-1033.1993.tb17986.x

[42] Thoma R, Loffler B, Stihle M, Huber W, Ruf A, Hennig M. Structural basis of proline-specific exopeptidase activity as observed in human dipeptidyl peptidase-IV. Structure. 2003;11(8):947–59. Epub 2003/08/09. S0969212603001606 [pii]. . 12906826 10.1016/s0969-2126(03)00160-6

[43] Sharma R, McDonald TS, Eng H, Limberakis C, Stevens BD, Patel S, et al In vitro metabolism of the glucagon-like peptide-1 (GLP-1)-derived metabolites GLP-1(9–36)amide and GLP-1(28–36)amide in mouse and human hepatocytes. Drug Metab Dispos. 2013;41(12):2148–57. Epub 2013/09/24. 10.1124/dmd.113.054254 dmd.113.054254 [pii]. . 24056839

[44] McDonald TJ, Perry MH, Peake RW, Pullan NJ, O'Connor J, Shields BM, et al EDTA improves stability of whole blood C-peptide and insulin to over 24 hours at room temperature. PLoS One. 2012;7(7):e42084 Epub 2012/08/04. 10.1371/journal.pone.0042084 PONE-D-12-06472 [pii]. 22860060 PMC3408407

[45] Sumpio BE, Riley JT, Dardik A. Cells in focus: endothelial cell. Int J Biochem Cell Biol. 2002;34(12):1508–12. Epub 2002/10/16. S1357272502000754 [pii]. . 12379270 10.1016/s1357-2725(02)00075-4

[46] Santoprete A, Capito E, Carrington PE, Pocai A, Finotto M, Langella A, et al DPP-IV-resistant, long-acting oxyntomodulin derivatives. J Pept Sci. 2011;17(4):270–80. Epub 2011/02/05. 10.1002/psc.1328 . 21294225

[47] Koomen JM, Li D, Xiao LC, Liu TC, Coombes KR, Abbruzzese J, et al Direct tandem mass spectrometry reveals limitations in protein profiling experiments for plasma biomarker discovery. J Proteome Res. 2005;4(3):972–81. Epub 2005/06/15. 10.1021/pr050046x . 15952745

[48] Blache P, Kervran A, Martinez J, Bataille D. Development of an oxyntomodulin/glicentin C-terminal radioimmunoassay using a "thiol-maleoyl" coupling method for preparing the immunogen. Anal Biochem. 1988;173(1):151–9. Epub 1988/08/15. 0003-2697(88)90172-8 [pii]. . 3189794 10.1016/0003-2697(88)90172-8

[49] Thim L, Moody AJ. The primary structure of porcine glicentin (proglucagon). Regul Pept. 1981;2(2):139–50. Epub 1981/05/01. . 6894800 10.1016/0167-0115(81)90007-0

[50] Rogatsky E, Stein DT. Two-dimensional reverse phase-reverse phase chromatography: A simple and robust platform for sensitive quantitative analysis of peptides by LC/MS. Hardware design. J Sep Sci. 2006;29(4):538–46. Epub 2006/04/06. . 16583692 10.1002/jssc.200500474

[51] van den Broek I, Niessen WM, van Dongen WD. Bioanalytical LC-MS/MS of protein-based biopharmaceuticals. J Chromatogr B Analyt Technol Biomed Life Sci. 2013;929:161–79. Epub 2013/05/21. 10.1016/j.jchromb.2013.04.030 S1570-0232(13)00241-9 [pii]. . 23685427

[52] Razavi M, Frick LE, LaMarr WA, Pope ME, Miller CA, Anderson NL, et al High-throughput SISCAPA quantitation of peptides from human plasma digests by ultrafast, liquid chromatography-free mass spectrometry. J Proteome Res. 2012;11(12):5642–9. Epub 2012/11/07. 10.1021/pr300652v . 23126378

[53] Zhang H, Xin B, Caporuscio C, Olah TV. Bioanalytical strategies for developing highly sensitive liquid chromatography/tandem mass spectrometry based methods for the peptide GLP-1 agonists in support of discovery PK/PD studies. Rapid Commun Mass Spectrom. 2011;25(22):3427–35. Epub 2011/10/18. 10.1002/rcm.5241 . 22002697

[54] Halquist MS, Sakagami M, Karnes HT. Determination of oxyntomodulin, an anorectic polypeptide, in rat plasma using 2D-LC-MS/MS coupled with ion pair chromatography. J Chromatogr B Analyt Technol Biomed Life Sci. 2012;903:102–11. Epub 2012/07/31. 10.1016/j.jchromb.2012.06.047 S1570-0232(12)00403-5 [pii]. . 22841744

[55] Miyachi A, Murase T, Yamada Y, Osonoi T, Harada K. Quantitative analytical method for determining the levels of gastric inhibitory polypeptides GIP1-42 and GIP3-42 in human plasma using LC-MS/MS/MS. J Proteome Res. 2013;12(6):2690–9. Epub 2013/04/24. 10.1021/pr400069f . 23607762

[56] Siskos AP, Katsila T, Balafas E, Kostomitsopoulos N, Tamvakopoulos C. Simultaneous absolute quantification of the glucose-dependent insulinotropic polypeptides GIP1-42 and GIP3-42 in mouse plasma by LC/ESI-MS/MS: preclinical evaluation of DP-IV inhibitors. J Proteome Res. 2009;8(7):3487–96. Epub 2009/05/12. 10.1021/pr900155h . 19425608

[57] Li YX, Hackman M, Wang CY. Quantitation of polypeptides (glucagon and salmon calcitonin) in plasma samples by 'high resolution' on a triple quadrupole mass spectrometer. Bioanalysis. 2012;4(6):685–91. Epub 2012/03/29. 10.4155/bio.12.12 . 22452259

