# Supplementary figures and images for: Degradation and Stabilization of Peptide Hormones in Human Blood Specimens
Source: PLoS One. 2015 Jul 29;10(7):e0134427. doi: 10.1371/journal.pone.0134427 (PMC4519045; doi:10.1371/journal.pone.0134427)

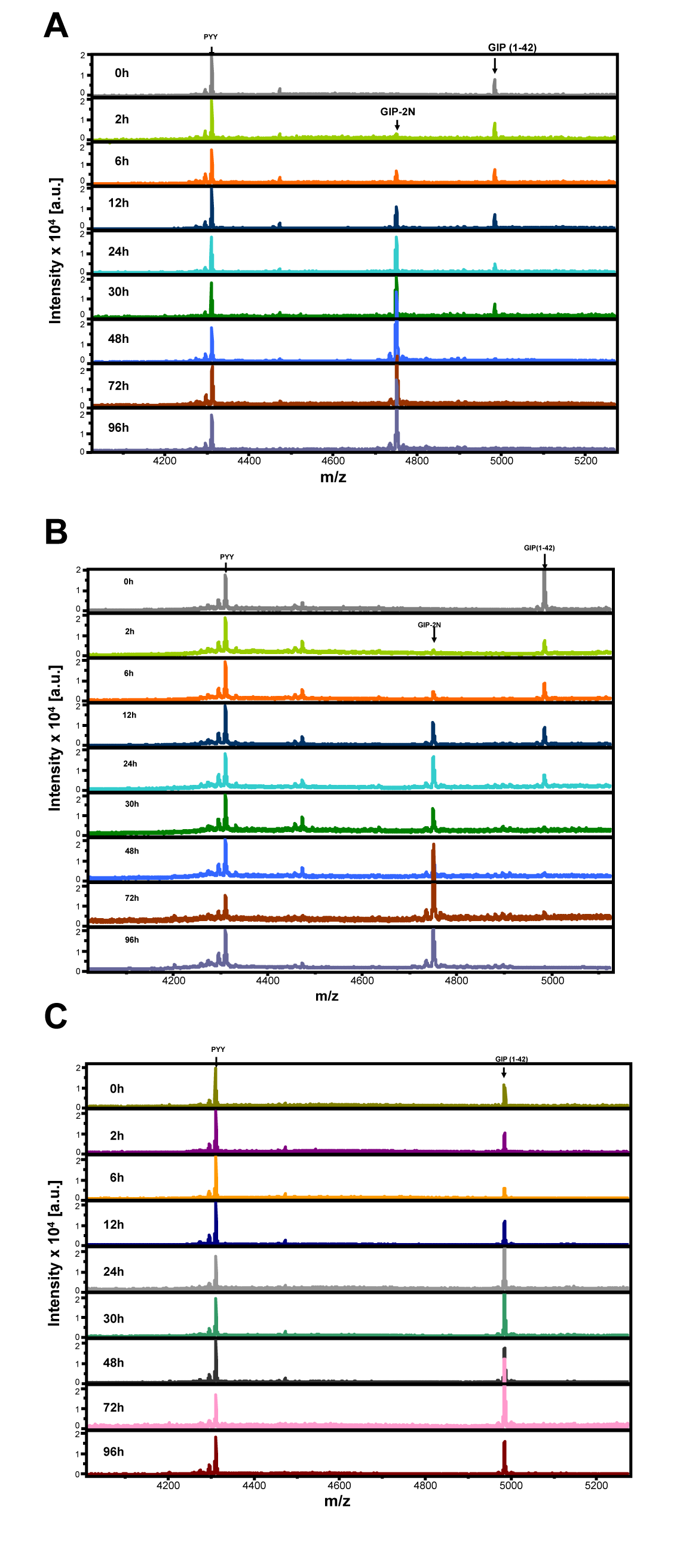

Supplement: S1 Fig — The peaks of GIP(1–42) and GIP(3–42) were indicated. The decrease of GIP(1–42) and increase of GIP(3–42) over incubation periods of time demonstrated that DPP-IV activity contributed to the degradation of GIP in the EDTA plasma (A) and serum (B) samples. The stable peak of GIP(1–42) was observed in the P800 plasma sample at room temperature. (C) Data are representative of 4 subjects with 1 or 2 replicates. (TIF) [file pone.0134427.s001.tif]

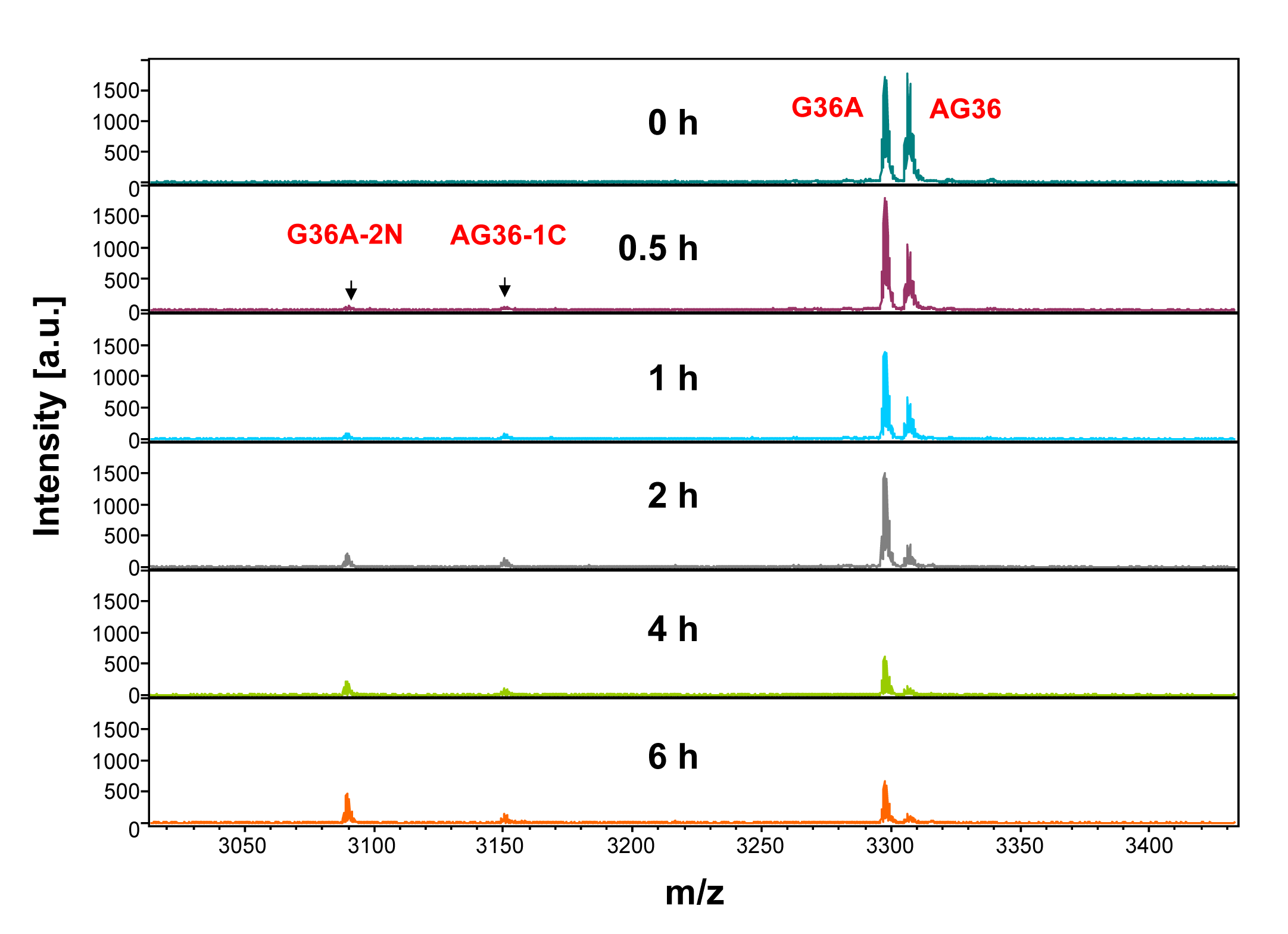

Supplement: S2 Fig — G36A and AG36 were spiked into and mixed with serum sample in 1:10 ratio (v/v). The sample was incubated at room temperature and aliquots were withdrawn for peptide extraction and MALD-TOF MS analysis at indicated time points. Both daughter peptides G36A-2N and G36-1C were detected after incubation for 30 minutes. Data are representative from 3 subjects tested in duplicate. (TIF) [file pone.0134427.s002.tif]

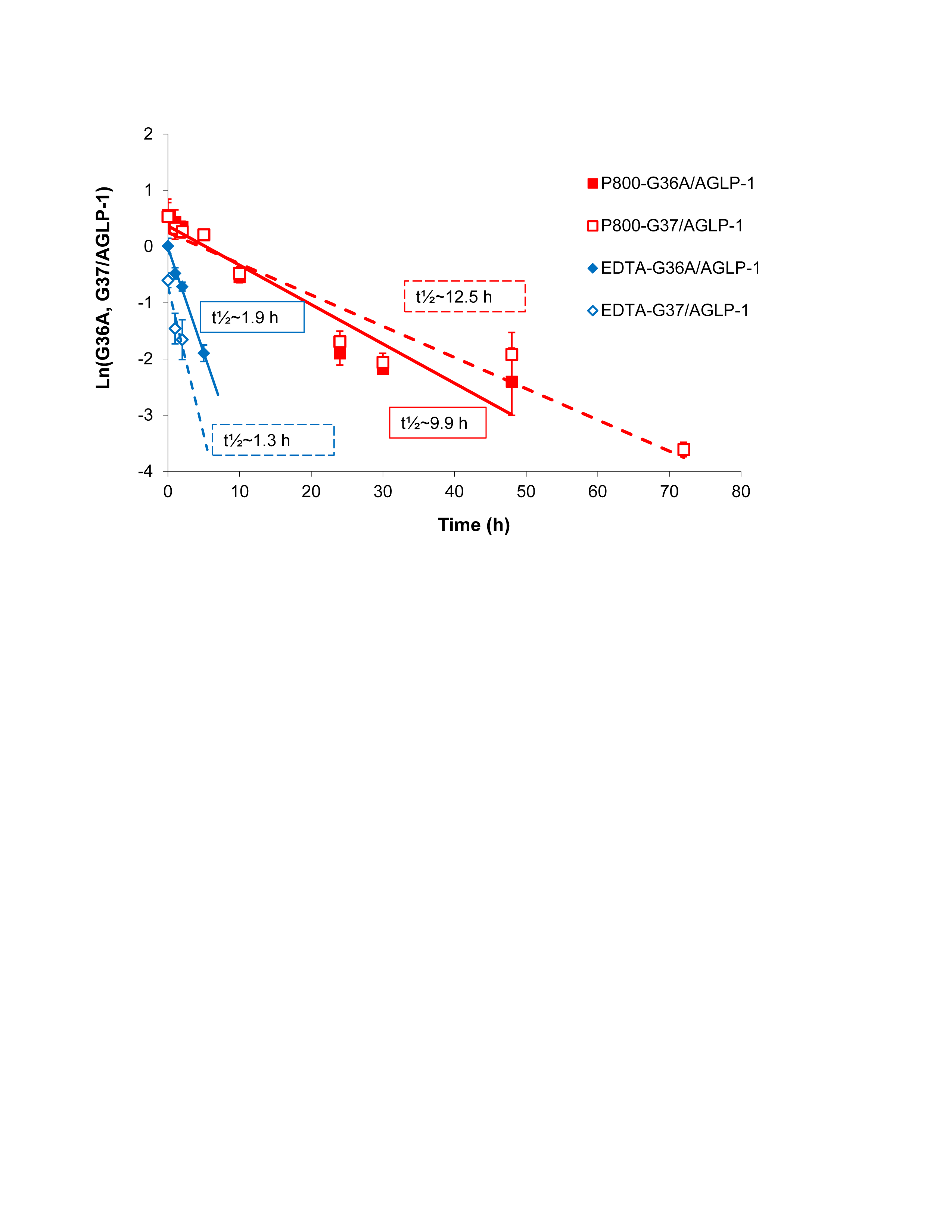

Supplement: S3 Fig — Two active GLP-1 forms (G36A and G37) were spiked into blood specimens freshly collected in EDTA and P800 tubes. The blood samples were incubated at room temperature for specified periods of time, centrifuged to collect plasma samples, followed by spiking AG36 as the internal control, and processed for MALDI-TOF MS analysis. The relative peak areas were plotted vs. the incubation time, and t½ of two peptides was determined. Data are from 3 subjects processed in duplicates. (TIF) [file pone.0134427.s003.tif]

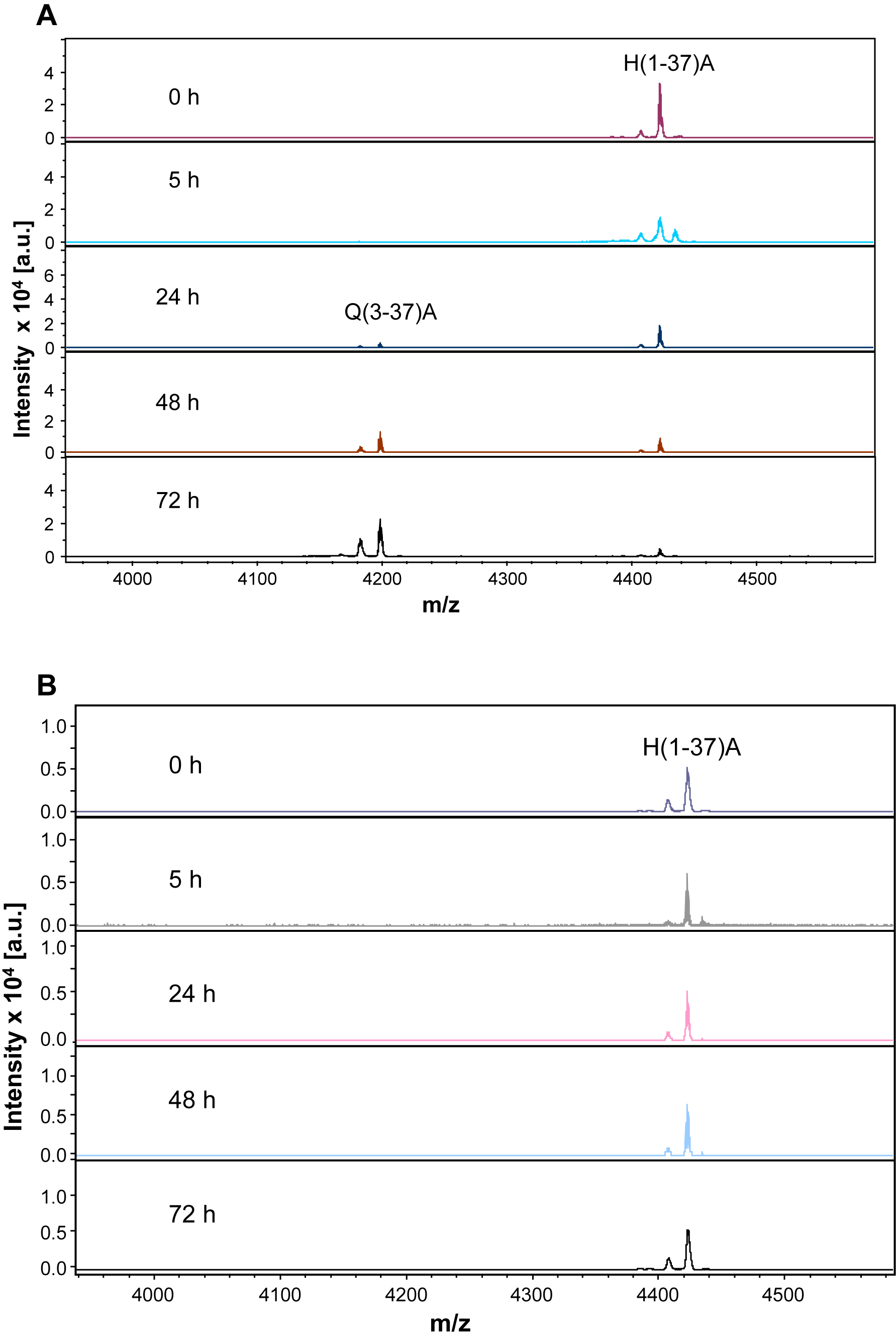

Supplement: S4 Fig — Time-course MS of OXM in EDTA plasma sample (A)EDTA plasma indicates its instability with the generation of OXM-2N. (B) P800 Plasma The preservation of OXM (1–37) was achieved for up to 72 hours. The data are representative from at least 6 subjects. (TIF) [file pone.0134427.s004.tif]

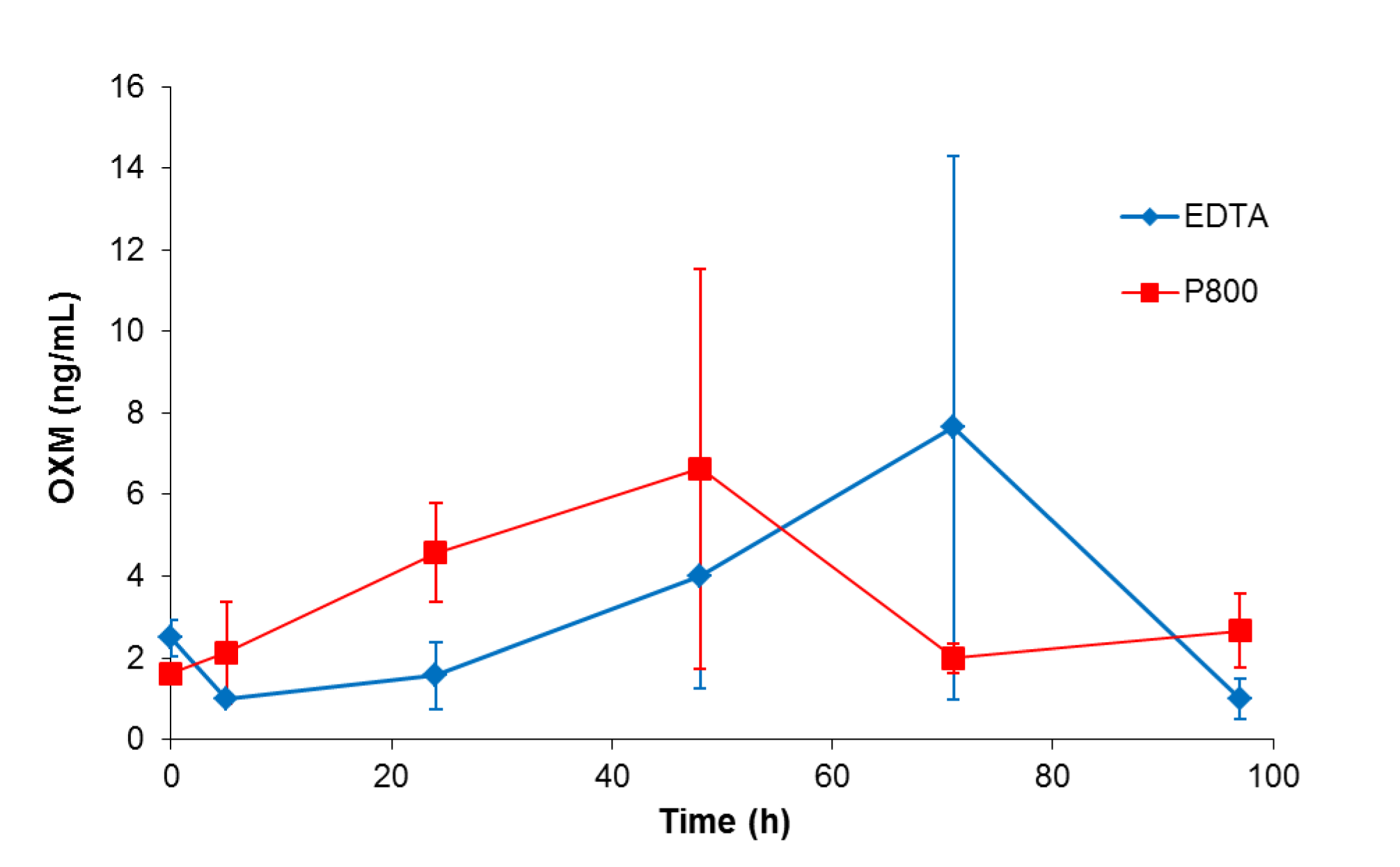

Supplement: S5 Fig — Time course was performed. (TIF) [file pone.0134427.s005.tif]

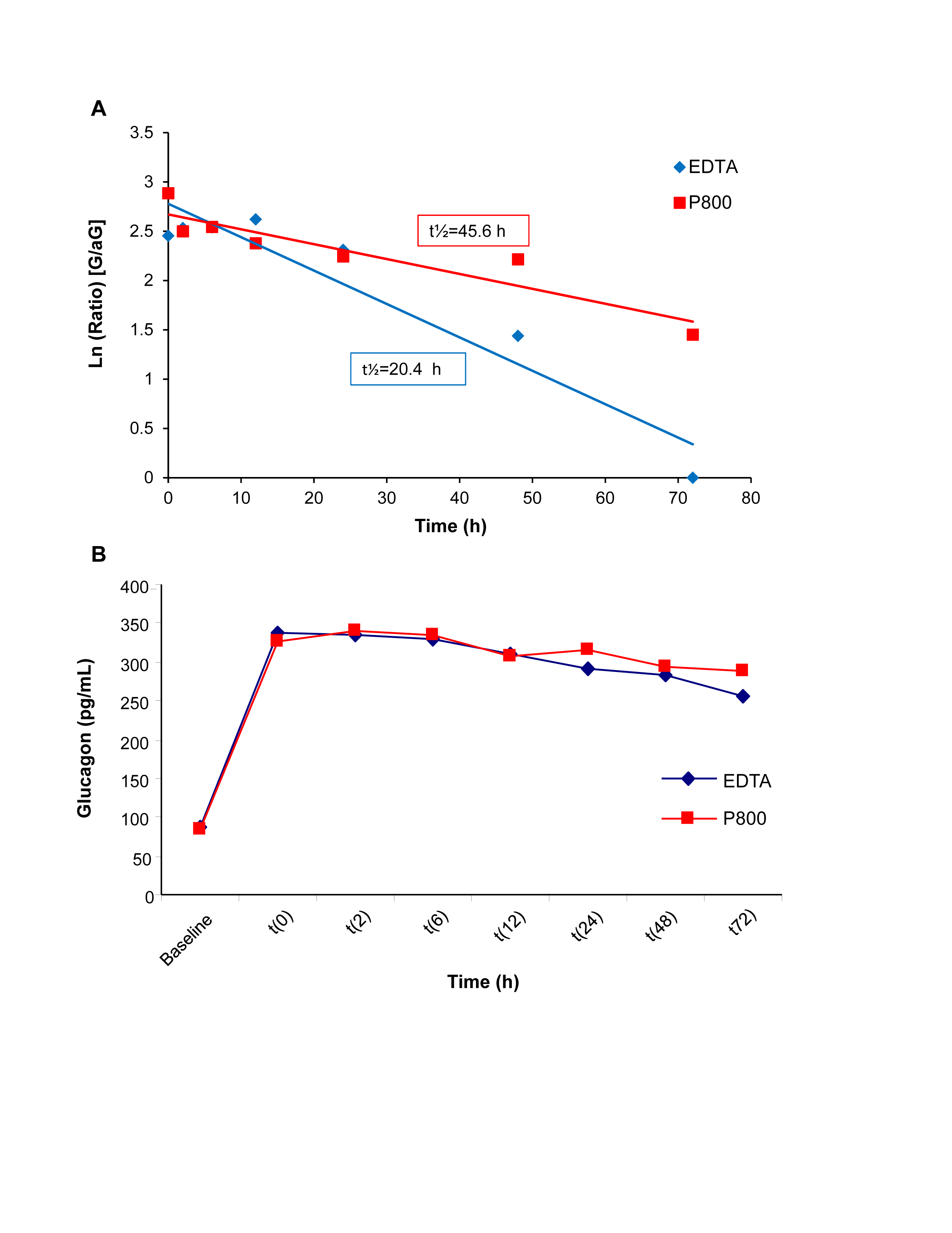

Supplement: S6 Fig — (A)Time-course MS (B) Time-course EIA. Data are from 1 subject in triplicate. (TIF) [file pone.0134427.s006.tif]
